# Synopsis of the Species of Coccidians Reported in Marine Fish

**DOI:** 10.3390/ani13132119

**Published:** 2023-06-26

**Authors:** Aurélia Saraiva, Jorge C. Eiras, Cristina Cruz, Raquel Xavier

**Affiliations:** 1 Department of Biology, Faculty of Sciences, University of Porto, Rua do Campo Alegre, Edifício FC4, 4169-007 Porto, Portugal; jceiras@fc.up.pt (J.C.E.);; 2 CIIMAR, Interdisciplinary Center of Marine and Environmental Research, Terminal de Cruzeiros do Porto de Leixões, Av. General Norton de Matos s/n, 4450-208 Matosinhos, Portugal; 3 CIBIO, Centro de Investigação em Biodiversidade e Recursos Genéticos, InBIO, Laboratório Associado, Campus Agrário de Vairão, Universidade do Porto, 4485-661 Vila do Conde, Portugal; 4 BIOPOLIS Program in Genomics, Biodiversity and Land Planning, CIBIO, Campus Agrário de Vairão, 4485-661 Vila do Conde, Portugal

**Keywords:** apicomplexa, eimeriorina, *Eimeria*, *Goussia*, *Epieimeria*, *Calyptospora*, *Crystallospora*, fish

## Abstract

**Simple Summary:**

Apicomplexa (Levine, 1970) are obligate parasites, of both vertebrate and invertebrate hosts, and may be responsible for important diseases. The majority of piscine apicomplexans are coccidians, which infect the cells of the alimentary tract and other extra-intestinal tissues. The goal of this work is to provide up-to-date information about the diversity of these parasites in marine fish. For this purpose, available information concerning Coccidia morphology and molecular profile, the hosts and the organs they infect, as well as their geographic distribution, was assembled. It is remarkable that from the 100 morphologically described species, only 6 have available genetic data.

**Abstract:**

Eimeriid coccidians represent one of the largest groups of parasitic unicellular organisms and comprise many species of veterinary and medical importance. The aim of this work is to provide information, as complete as possible, concerning the oocyst phase of the species of coccidians (Apicomplexa: Eimeriorina) with four sporocysts, which were reported in marine fish. For each species, the accepted scientific name and respective authorities, its synonyms, reported hosts, site of infection (organ), and geographic distribution have been assembled. Available information on morphology (oocyst, sporocyst, and sporozoite) and GenBank accession numbers were also compiled. A total of 100 species of coccidians were described and reported from 60 families of marine fishes. Most species have been described from marine teleosts, with only 14 of the species described from marine elasmobranchs. Most of the species reported in marine fish belong to the genera *Eimeria* and *Goussia*, and only a handful belong to the genera *Epieimeria*, *Calyptospora*, and *Crystallospora*. Although marine coccidians have began to be described for more than one century, the collection of genetic data on marine fish coccidians only started in the 2010s and remains largely disconnected from the morphological analysis of specimens, which is available for only six formally described species.

## 1. Introduction

Eimeriid coccidians represent one of the largest groups of parasitic unicellular organisms and comprise many species of veterinary and medical importance [1]. Although the first fish coccidian studies were conducted in the last decade of the nineteenth century, still, little is known about the life cycles, morphology, phylogenetic, and infection patterns of these parasites, especially those that occur in marine fish.

Coccidians infecting marine fish belong to the genera *Eimeria* Schneider, 1875; *Goussia* Labbé, 1896; *Epieimeria* Dyková and Lom, 1981; *Calyptospora* Overstreet, Hawkins and Fournie, 1984; and *Crystallospora* Labbé, 1896. It should be stressed that the genus *Goussia* was erected to accommodate piscine coccidians with oocysts possessing four sporocysts, with two valves joined by a longitudinal suture. This genus has a controversial history, but the studies conducted in the last decades indicate that *Goussia* is a valid genus, which seems to represent the ancestral state of the Sarcocystidae, Eimeriidae, and Calyptosporidae [2,3,4]. Importantly, Benajiba et al. [5] suppressed the genus *Epieimeria* Dyková and Lom, 1981, on the basis of the intracellular gamogony, merogony, and sporogony of the type species *E. anguillae*. Observations conducted by Sitjà-Bobadilla et al. [6] further support the suppression of this genus. If these features are confirmed in all described species of the genus *Epieimeria*, they should be moved to the genus *Eimeria*. 

Adding to a troubled taxonomy, the information on coccidians that infect marine fish is currently widely dispersed across both older and more recent scientific publications and, apart from the annotated list provided in the work of Dyková and Lom [7] from the early 1980s, no other attempt has been made to review all the information on fish coccidians. The aim of this work is to compile, as complete as possible and for the first time, the available information concerning the oocyst phase of the species of coccidians (Apicomplexa: Eimeriorina) with four sporocysts which has been reported from marine fish.

The specific objectives of this review were to provide: (i) an up-to-date list of species of marine piscine coccidians; (ii) a complete overview of their known hosts, geographic range, and infected organs; (iii) morphological characterization of each species and, whenever possible, drawings of their oocysts; (iv) a summary of their phylogenetic affinities; and (v) a discussion of the state of the art, identifying knowledge gaps and future research areas. 

## 2. Materials and Methods

A bibliographic search was performed using all available databases and fields included on Web of Science (https://www.webofscience.com/wos/woscc/basic-search) with the following search string: (Coccid* or *Goussia* or *Eimeria* or *Epieimeria* or *Crystallospora* or *Calyptospora*) and fish.

For the purpose of constructing a list of species infecting marine fish, species that were given a generic but not specific assignations were not considered. Additionally, species for which a Stieda body was not referred, but that were still reported in genus *Eimeria*, were maintained in this genus until new data are available.

For each coccidian species, we assembled the accepted scientific name and respective authorities, eventual synonyms (syn), hosts, site of infection (organ), and geographic distribution. Regarding the geographic distribution, the precision of the available information in the reviewed manuscripts varied significantly (in some cases, a country was mentioned; in others, an ocean; and in others, a very specific location on the coast of a country). In cases for which sea(s) or ocean(s) were not referenced in the original publication, this information was added. 

Available information on morphology and dimensions (oocyst, sporocyst, and sporozoite) is detailed according to what was reported in the reviewed publications. GenBank accession numbers, whenever available, were also compiled. Additionally, any other information that was deemed important was also supplied. Hosts’ family and names (both vernacular and scientific names) were reported according to FishBase [8]. 

**Figure 1:**
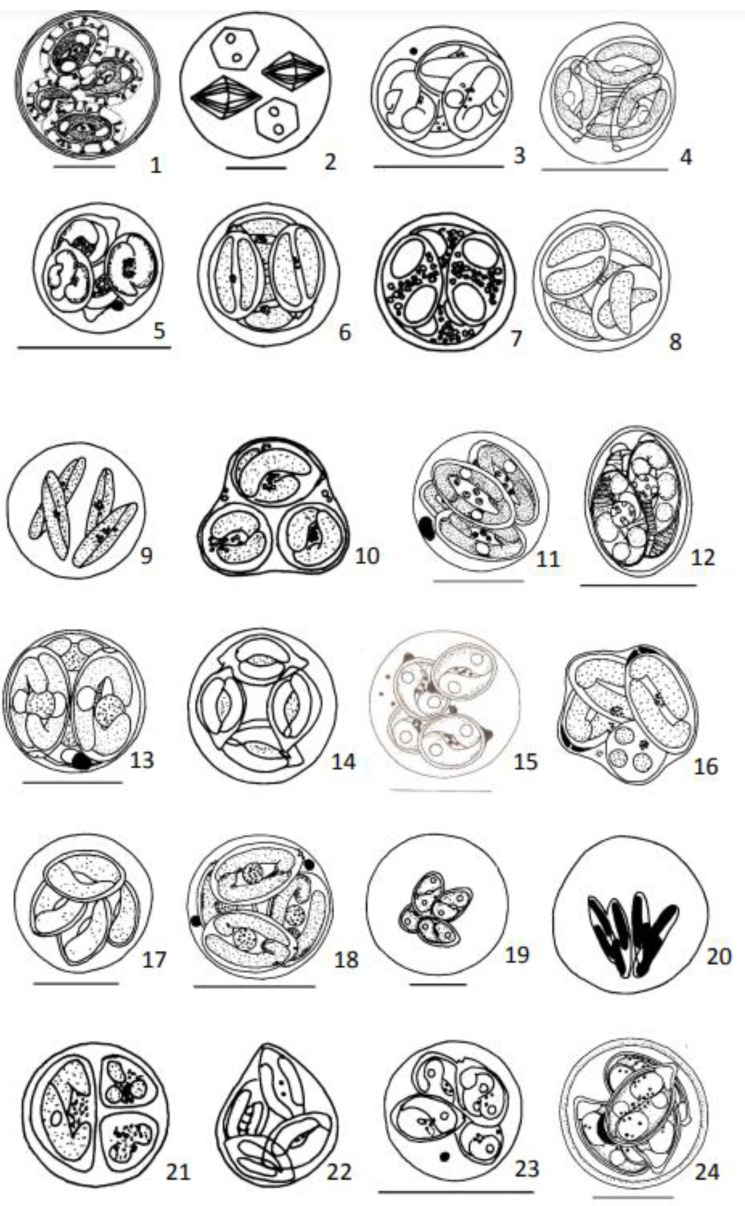
Diagrammatic illustrations of coccidian’s oocysts redrawn from publications referred after species name. 1—*Calytospora funduli* from Duszynski et al., 1979; 2—*Crystallospora crystalloides* from Lom and Dyková, 1992; 3—*Eimeria adioryxi* from Diouf and Toguebaye, 1994; 4—*Eimeria anguillae* from Hine, 1975; 5—*Eimeria ashburneri* from Diouf and Toguebaye, 1994; 6—*Eimeria atherinae* from Daoudi et al., 1987; 7—*Eimeria banyulensis* from Lom and Dyková, 1982; 8—*Eimeria bouixi* from Daoudi and Marquês, 1986; 9—*Eimeria brevoortiana* from Hardcastle, 1944; 10—*Eimeria catalana* from Lom and Dyková, 1981; 11—*Eimeria cheilodactyli* from Molnár and Rodhe, 1988; 12—*Eimeria chollaensis* from Upton et al., 1988; 13—*Eimeria ciliatae* from Molnar and Rodhe, 1988; 14—*Eimeria citriformis* from Bykhovskaya-Pavlovskaya et al., 1964; 15—*Eimeria dakarensis* from Diouf and Toguebaye, 1994; 16—*Eimeria dicentrarchi* from Daoudi and Marquês, 1986; 17—*Eimeria dingleyi* from Davies, 1978; 18—*Eimeria dykovae* from Molnár and Rodhe, 1988; 19—*Eimeria ethmalosae* from Diouf and Toguebaye, 1994; 20—*Eimeria etrumei* from Bykhovskaya-Pavlovskaya et al., 1964; 21—*Eimeria euzeti* from Daoudi et al., 1987; 22—*Eimeria evaginata* from Bykhovskaya-Pavlovskaya et al., 1964; 23—*Eimeria gabonensis* from Diouf and Toguebaye, 1994; 24—*Eimeria halleri* from Upton et al., 1986. Scale bar = 10 µm. Figure 1(6–10, 14, 16, 20–22) and Figure 2(25) are not to scale.

**Figure 2:**
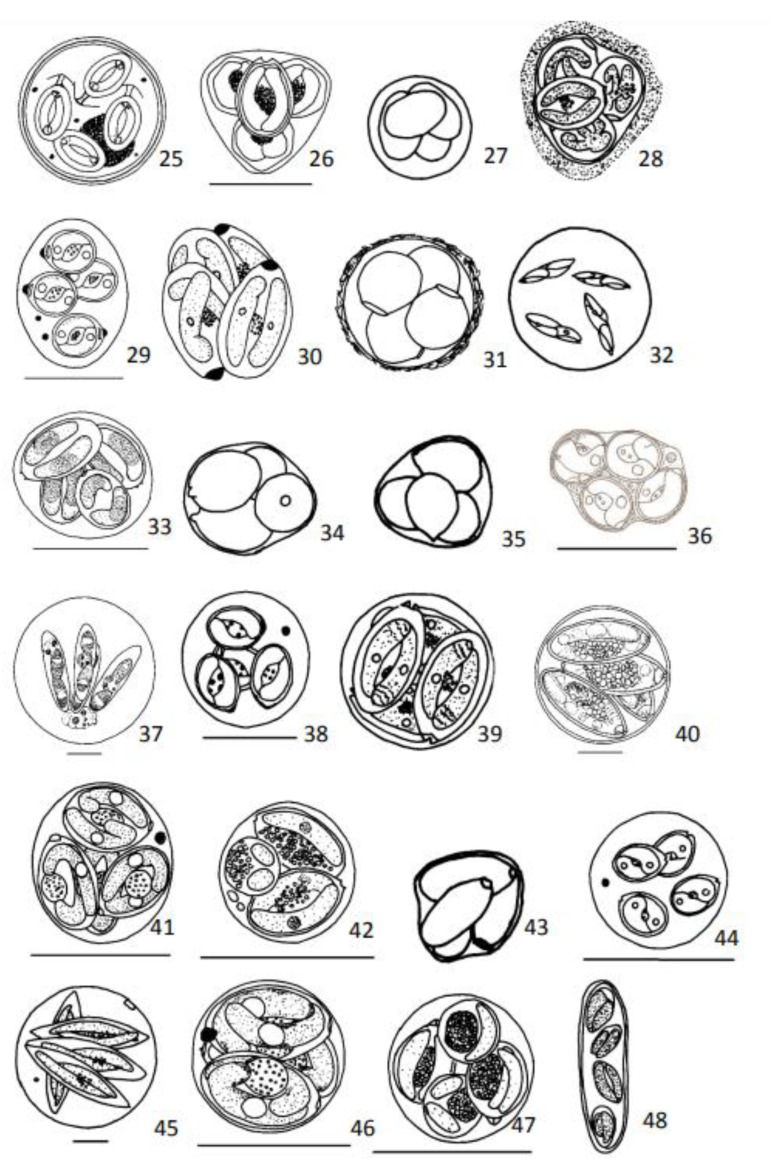
Diagrammatic illustrations of coccidian’s oocysts redrawn from publications referred after species name. 25—*Eimeria harpodoni* from Setna and Bana, 1935; 26—*Eimeria hexagona* from Lom and Dyková, 1992; 27—*Eimeria insignis* from Lom and Dyková, 1982; 28—*Eimeria ivanae* from Lom and Dyková, 1981; 29—*Eimeria kayarensis* from Diouf and Toguebaye, 1994; 30—*Eimeria kotorensis* from Daoudi et al., 1987; 31—*Eimeria lairdi* from Lom and Dyková, 1981; 32—*Eimeria myoxocephali* from Fitzgerald, 1975; 33—*Eimeria nesowai* from Lom and Dyková, 1995; 34—*Eimeria nucleocola* from Lom and Dyková, 1981; 35—*Eimeria ottojiroveci* from Lom and Dyková, 1981; 36—*Eimeria palavensis* Marquès and Capapé, 2001; 37—*Eimeria patagonensis* from Timi and Sardella, 1998; 38—*Eimeria perciformis* from Diouf and Toguebaye, 1994; 39—*Eimeria petrovici* from Daoudi et al.,1987; 40—*Eimeria phyllopterycis* from Upton et al., 2000; 41—*Eimeria pleurostici* from Molnar and Rodhe, 1988; 42—*Eimeria rohdei* from Lom and Dyková, 1995; 43—*Eimeria roussillona* from Lom and Dyková, 1981; 44—*Eimeria ryptici* from Diouf and Toguebaye, 1994; 45—*Eimeria sardinae* from Lom and Dyková, 1992; 46—*Eimeria sillaginis* from Molnár and Rodhe, 1988; 47—*Eimeria smaris* from Daoudi et al., 1989; 48—*Eimeria southwelli* from Halawani, 1930. Scale bar = 10 µm. Figure 2(27, 28, 30, 31, 32, 34, 35, 39, 43, and 48) are not to scale.

**Figure 3:**
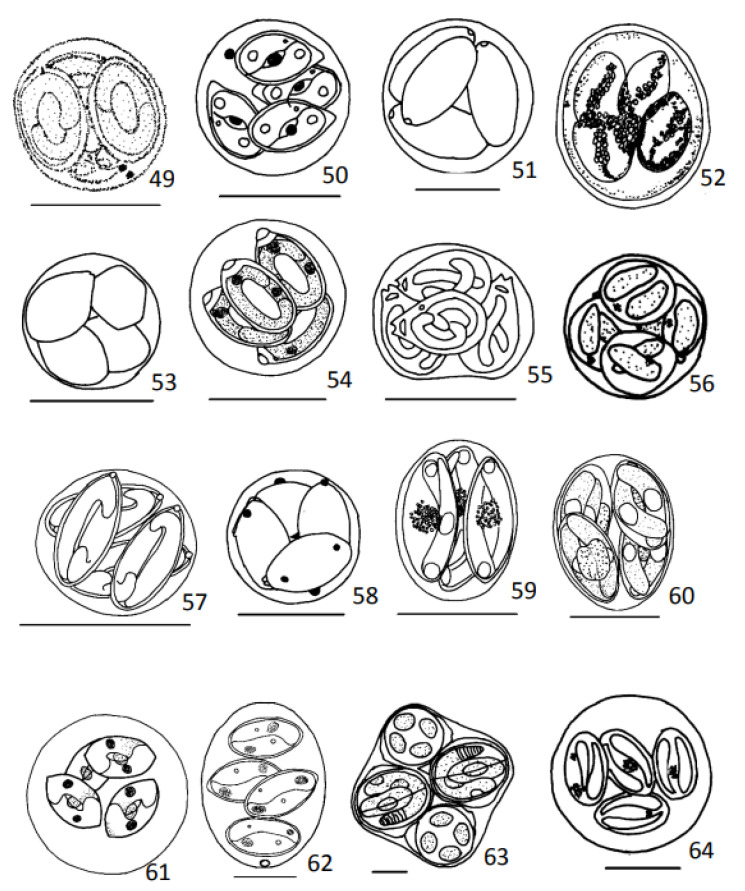
Diagrammatic illustrations of coccidian’s oocysts redrawn from publications referred after species name. 49—*Eimeria sparis* from Sitja-Bobadilla et al., 1996; 50—*Eimeria syacii* from Diouf and Toguebaye, 1994; 51—*Eimeria symphodi* from Daoudi et al. 1989a; 52—*Eimeria syngnathi* from Bykhovskaya-Pavlovskaya et al., 1964; 53—*Eimeria triglae* from Daoudi et al., 1989; 54—*Eimeria variabilis* from Lom and Dyková, 1992; 55—*Epieimeria isabellae* from Lom and Dyková, 1992; 56—*Epieimeria lomae* from Daoudi et al., 1987; 57—*Epieimeria ocellata* from Landsberg, 1993; 58—*Epieimeria puytoraci* from Daoudi et al., 1989a; 59—*Goussia aculeati* from Lom and Dyková, 1992; 60—*Goussia arrawarra* from Molnár and Rodhe, 1988; 61—*Goussia auxidis* from Jones, 1990; 62—*Goussia bayae* from Matsche et al., 2019; 63—*Goussia caseosa* from Lom and Dyková, 1992; 64—*Goussia clupearum* from Lom and Dyková, 1992. Scale bar = 10 µm. Figure 3(52, 56, and 61) are not to scale.

**Figure 4:**
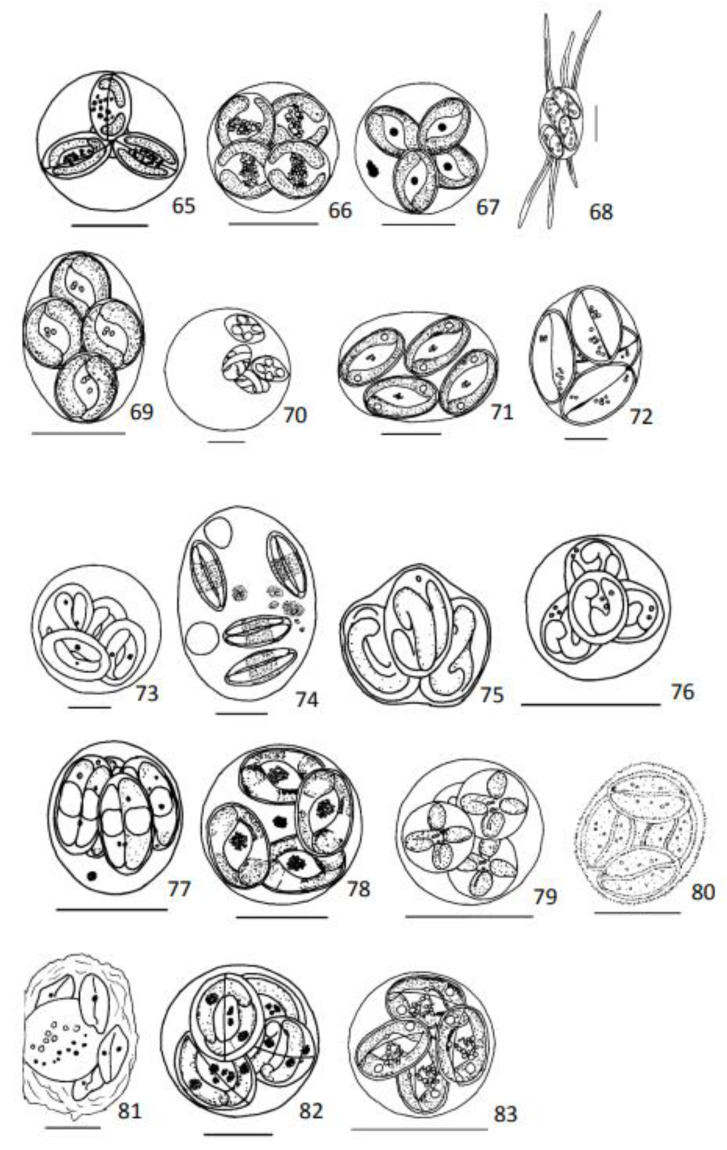
Diagrammatic illustrations of coccidian’s oocysts redrawn from publications referred after species name 65—*Goussia cruciata* from Lom and Dyková, 1995; 66—*Goussia dakarensis* from Diouf and Toguebaye, 1993; 67—*Goussia decapteri* from Diouf and Toguebaye, 1993; 68—*Goussia echinata* from Friend, Lovy and Hershberger, 2016; 69—*Goussia emissolei* from Diuof and Toguebaye, 1993; 70—*Goussia ethmalotis* from Obiekezie (1986); 71—*Goussia exoceti* from Diouf and Toguebaye, 1993; 72—*Goussia floridana* Landsberg, 1993; 73—*Goussia gadi* from Lom and Dyková, 1992; 74—*Goussia kuehae* from Székely et al., 2013; 75—*Goussia luciae* from Lom and Dyková, 1982; 76—*Goussia lucida* from Lom and Dyková, 1992; 77—*Goussia microcanthi* from Molnár and Rodhe, 1988; 78—*Goussia senegalensis* from Diouf and Toguebaye, 1993; 79—*Goussia soumbediounensis* from Diouf and Toguebaye, 1993; 80—*Goussia sparis* from Sitja-Bobadilla et al., 1996; 81—*Goussia squali* from Lom and Dyková, 1992; 82—*Goussia thelohani* from Lom and Dyková, 1995; 83—*Goussia trachinoti* from Diouf and Taguebaye, 1993. Scale bar = 10 µm. Figure 4(75) is not to scale.

Measurements, unless otherwise stated, are provided in µm. Oocysts were redrawn whenever good drawings were available in the literature (Figure 1, Figure 2, Figure 3 and Figure 4). In all presented drawings, scale bars are adapted to equal to 10 µm. Some drawings are not to scale. 

Additionally, genetic data available on NCBI’s GenBank (https://www.ncbi.nlm.nih.gov/genbank/) for the 18S rRNA region of piscine coccidians, including those of lineages recovered from marine fish which were not identified to the species level, were compiled for phylogenetic analysis. Based on previously published phylogenies and the recognized existence of different genetic groups [4], as well as results from BLAST (Basic Local Alignment Tool) analysis, five different phylogenetic trees were reconstructed using Bayesian inference, implemented in MrBayes v3.2.6 [9]. Sequence alignment was performed using MAFFT software [10], and sequence models of evolution were selected using jModelTest 2.1.6 [10]. Based on the BLAST analysis of the sequences from ingroups, either *Isospora belli* (DQ060661) or *Intranuclear coccidium* (AY728896) were selected as outgroups. Three available sequences were not considered due to their short length, all belonging to *Goussia clupearum* (MF468314, MF468308 and MT463285).

## 3. Results

Our search resulted in 100 species of coccidians, detected in 60 families of marine fishes (Table 1), and distributed among 5 genera: *Calyptospora* and *Crystallospora*, both with 1 described species each; *Epieimeria*, with 4 described species; *Eimeria*, with 64 species; and *Goussia*, with 30 species. For some species, it was not possible to obtain all information concerning the characteristics taken into account for this review. 

### 3.1. Species List

***Calyptospora funduli*** (Duszynski, Solangi and Overstreet, 1979) Overstreet, Hawkings and Fournie, 1984

Syn: *Eimeria funduli* Duszynski, Solangi and Overstreet, 1979.

Host: Gulf killifish, *Fundulus grandis*, Fundulidae; Saltmarsh topminnow, *Fundulus jenkinsi*, Fundulidae; Bayou killifish, *Fundulus pulvereus*, Fundulidae; Longnose killifish, *Fundulus similis*, Fundulidae; Mummichog, *Fundulus heteroclitus*, Fundulidae; Inland silverside, *Menidia beryllina*, Atherinopsidae; gulf toadfish, *Opsanus beta*, Batrachoididae.

Locality: Off US and Gulf of Mexico, Atlantic Ocean.

Organ: Liver, pancreas, occasionally fat tissue, mesentery, ovary, intestine, gall bladder and dermis.

Oocysts: Spherical, 25 (20–31), without residuum (Figure 1(1)).

Sporocysts: Ovoid 10 (9–11) × 6 (5–7), bearing 10 to 25 sporopodia that support a veil, one to four small refractile granular residua.

Sporozoites: With one large refractile body near posterior end.

GenBank accession number: 18S rRNA: GU479670, FJ904646, FJ904645, FJ904644, FJ904643.

Infection cannot be transmitted directly by oocysts. Crustaceans (*Palaemonetes* and *Macrobrachium* species) act as intermediate hosts.

In heavy infections, large aggregations of oocysts replaced up to 85% of the liver. Heavy infections have a detrimental effect on hosts. 

References: [11,12,13,14]

***Crystallospora crystalloides*** (Thélohan, 1893) Labbé, 1899

Syn: *Coccidium crystalloides*, Thélohan, 1893; *Crystallospora thelohani*, Labbé, 1896; *Eimeria crystalloides*, (Thélohan, 1983) Doflein, 1909.

Host: Shore rockling, *Gaidropsarus mediterraneus* (referred as *Motella fusca*), Gaidropsaridae; Three-bearded rockling, *Gaidropsarus vulgaris* (referred as *Motella maculate* and *M. tricirrata*), Gaidropsaridae.

Locality: Atlantic Sea; Mediterranean Sea.

Organ: Intestine.

Oocyst: Round, 20–24 (Figure 1(2)).

Sporocysts: 15 × 9.5.

References: [7,13,15]

***Eimeria adioryxi*** Diouf and Toguebaye, 1994

Host: Red squirrelfish, *Sargocentron hastatum* (referred as *Adioryxi hastatus*), Holocentridae.

Locality: Off Senegal, Atlantic Ocean.

Organ: Intestine.

Oocysts: Spherical, 9.7 ± 0.6, without residuum (Figure 1(3)).

Sporocysts: Ovoid, 6.8 ± 0.8 long and 5.1 ± 0.3 wide, with Stieda body, four refringent granular residua; Sporozoites: Forming a cross.

References: [16]

***Eimeria anguillae*** Léger and Holland, 1992

Syn: *Epieimeria anguillae,* (Léger and Holland, 1922) Dyková and Lom, 1981.

Host: European eel, *Anguilla anguilla*, Anguillidae; New Zealand longfin eel, *Anguilla dieffenbachiii*, Anguillidae; short-finned eel, *Anguilla australis*, Anguillidae; American eel, *Anguilla rostrata*, Anguillidae.

Locality: Languedoc and Camargue lagoons, Mediterranean Sea; Adriatic Sea.

Organ: Intestine.

Oocysts: Spherical, 8.0–12.8 (Figure 1(4)).

Sporocysts: Ellipsoidal, 5.6–8.8 × 2.4–5.6, hexagonal in transverse section, with Stieda body and globular residuum.

Sporozoites: Curved.

GenBank accession number: 18S rRNA: GU479633, GU593704.

These parasites can cause mucosa and submucosa degeneration, emaciation and death. 

References: [5,7,13,17,18,19,20]

***Eimeria ashburneri*** Molnár and Rodhe, 1988

Host: Red Pandora, *Pagellus bellottii*, Sparidae; bluespotted seabream, *Pagrus caeruleostictus* (referred as *Sparus caeruleostictus*), Sparidae.

Locality: Off Senegal, Atlantic Ocean.

Organ: Intestine.

Oocysts: Spherical 8.5 ± 0.6, without residuum (Figure 1(5)).

Sporocysts: Ovoid, 5.9 ± 0.6 long by 3.9 ± 0.4 wide, with Stieda body, small granular residuum.

Sporozoites: Falciform, two per sporocysts arranged in cross.

Molnár and Rohde [21] detected this species in freshwater fish, Golden perch, *Macquaria ambigua*, Percichthydae.

References: [16]

***Eimeria atherinae*** Daoudi, Radujkovic, Marquès and Bouix, 1987

Host: Big-scale sand smelt, *Atherina boyeri*, Atherinidae.

Locality: Kotor Bay, Adriatic Sea; Thau pond, Mediterranean Sea.

Organ: Intestine.

Oocysts: Spherical 12 (11.5–13.5), without residuum (Figure 1(6)).

Sporocysts: Ellipsoidal 8.0 (7.0–9.0) × 5.0 (4.5–5.5) with Stieda body (in drawings not visible), granular residuum.

Sporozoites: 7.0 × 1.5.

References: [19,20]

***Eimeria banyulensis*** Lom and Dyková, 1982

Host: Axillary wrasse, *Symphodus mediterraneus* (referred as *Crenilabrus mediterraneus*), Labridae; Grey wrasse, *Symphodus cinereus*, Labridae.

Locality: Banyuls-sur-Mer, Mediterranean Sea; Adriatic Sea.

Organ: Intestine.

Oocysts: Spherical, 6.5–8.5), without residuum (Figure 1(7)).

Sporocysts: Ellipsoidal, 4.5–5.5 × 3.0–4.0, without Stieda body, with a mass of granular residuum.

Sporozoites: Ellipsoidal 1.5–2.0.

References: [7,20,22,23]

***Eimeria bouixi*** Daoudi and Marquès, 1986

Host: European seabass, *Dicentrarchus labrax*, Moronidae.

Locality: Mediterranean Sea.

Organ: Pyloric caeca.

Oocysts: Spherical, 7.0 (6.0–7.6) (Figure 1(8)).

Sporocysts: Ellipsoidal, 4.4 (4.0–4.9) × 3.0 (2.6–3.2) without Stieda body.

Sporozoites: 3.0–1.0.

According to the authors all developing stages are intra-nuclear. 

References: [24]

***Eimeria brevoortiana*** Hardcastle, 1944

Host: Atlantic menhaden, *Brevoortia tyrannus*, Clupeidae.

Locality: US coast of North Carolina, Atlantic Ocean.

Organ: Pyloric caeca, testes.

Oocysts: Spherical 17.0–30.0 or ellipsoidal 15.0–22.0 × 26.0–30.0), with residuum (Figure 1(9)).

Sporocysts: Elongate, 16.0 × 6.0, with residuum; no information of Stieda body.

Sporozoites: Unflexed.

Reference: [7,13,25]

***Eimeria catalana*** Lom and Dyková, 1981

Host: Axillary wrasse, *Symphodus mediterraneus* (referred as *Crenilabrus mediterraneus*), Labridae; grey wrasse, *Symphodus cinereus*, Labridae; blackbar hogfish, *Bodianus speciosus*, Labridae; Guinean parrotfish, *Scarus hoefleri*, Scaridae.

Locality: Banyuls-sur-Mer, Mediterranean Sea; Adriatic Sea; off Senegal, Atlantic Ocean.

Organ: Intestine.

Oocysts: About eleven residua in the form of a few refractile granules (Figure 1(10)).

Sporocysts: Ovoid, 7.5–8.5 × 5.5–6.5, with a narrower end bearing a flat ring-like thickening around Stieda body; granular residuum. 

Sporozoites: C shaped and with flexed ends.

Reference: [7,13,20,23,26]

***Eimeria cheilodactyli*** Molnár and Rodhe, 1988

Host: Red morwong, *Morwong fuscus* (referred as *Cheilodactylus fuscus*), Latridae.

Locality: Tasmania Sea.

Organ: Pyloric caeca, intestine.

Oocysts: Round, 11.2 (10.5–11.5), without residuum (Figure 1(11)).

Sporocysts: Elongate ellipsoidal, 9.6 (8.6–10.4) × 4.2 (4.0–4.4), Stieda body small, scattered coarse of granular residuum.

Sporozoites: Vermiform with one end reflexed.

References: [27]

***Eimeria chollaensis*** Upton, Garder and Duszynski, 1988

Host: Haller’s round ray (=round stingray), *Urobatis halleri* (=*Urolophus halleri*), Urotrygonidae.

Locality: Gulf of California, Pacific Ocean.

Organ: Spiral valve.

Oocysts: Ovoid, 13.3 (11.2–16.0) × 9.7 (8.0–10.8), lenght/width ratio 1.4 (1.2–1.5) (Figure 1(12)).

Sporocysts: Ovoid, 8.9 (8.0–10.0) × 4.9 (4.2–5.6), lenght/width ratio 1.8 (1.6–2.1), with Stieda but no substieda body, compact spherical residuum, 2.5 (2.0–3.4).

Sporozoites: Elongate, arranged head to tail within the sporocyst, with numerous transverse striations anteriorly and with a single, ovoid refractile body.

Sporulation exogenous. All oocysts recovered unsporulated from faeces.

Reference: [28,29]

***Eimeria ciliatae*** Molnár and Rodhe, 1988

Host: Sand sillago, *Sillago ciliata* (referred as sand whiting), Sillaginidae.

Locality: Tasmania Sea.

Organ: Pyloric caeca, intestine.

Oocysts: Round, 14.0 (13.4–14.2), without residuum (Figure 1(13)).

Sporocysts: Oval, 11.2 (10.9–11.7) × 7.3 (6.7–7.6), with small thickenings and cap on tapered end, round and finely granular residuum.

Sporozoites: Vermiform.

References: [21]

***Eimeria citriformis*** Dogiel, 1948

Host: *Tilesina gibbosa*, Agonidae.

Locality: Sea of Japan.

Organ: Pyloric caeca.

Oocysts: Spherical, 15 (Figure 1(14)).

Sporocysts: Lemon-like, 8.0–9.0 × 5.0.

Reference: [7,30]

***Eimeria clini*** Fantham, 1932

Host: Clinus superciliosus, Clinidae.

Locality: Off South Africa (Atlantic Ocean or Indian Ocean?). 

Organ: Intestine.

We were unable to obtain the article describing this species or any subsequent one.

Reference: [7]

***Eimeria dakarensis*** Faye, 1988

Host: Bluespotted seabass, *Cephalopholis taeniops*, Serranidae; Blacktip grouper, *Epinephelus fasciatus* (referred as *Epinephelus alexandrinus*), Serranidae.

Locality: Off Senegal, Atlantic Ocean.

Organ: Pyloric caeca, intestine.

Oocysts: Spherical, 11.4 ± 0.7, without residuum (Figure 1(15)).

Sporocysts: Pear-shaped 6.8 ± 0.6 long by 4.6 ± 0.9 wide, with Stieda body, four refringent granular residua.

References: [16]

***Eimeria dicentrarchi*** Daoudi and Marquès, 1986

Host: European seabass, *Dicentrarchus labrax*, Moronidae.

Locality: Languedoc, Mediterranean Sea; Adriatic Sea.

Organ: Pyloric caeca, intestine. 

Oocysts: Spherical, 11.2 (10.6–12.2) (Figure 1(16)).

Sporocysts: Ellipsoidal, 6.8–8.6 × 4.2–5.6, with a flattened, split-like Stieda body, four to five dispersed granular residua.

Sporozoites: Banana shaped, 4.8–6.9 × 1.4–2.0.

References: [24,31]

***Eimeria dingleyi*** Davies, 1978

Host: Shanny, *Lipophryus pholis* (referred as *Blennius pholis*), Bleenniidae.

Locality: Saint George Channel, Welsh coast, Atlantic Ocean.

Organ: Intestine.

Oocysts: Spherical, 16.1–19.2 to subspherical, 18.8–20.0 × 13.9–14.2, without residuum (Figure 1(17)).

Sporocysts: Spherical to ellipsoid 9.4–9.9 × 5.8–6.1, without Stieda body, without residuum.

Sporozoites: 8.6–9.0 × 2.6–3.1, single large refractile body at broad end.

Reference: [7,32]

***Eimeria dogieli*** (Dogiel, 1948) Pellérdy, 1963

Syn: *E. sphaerica* Dogiel, 1948.

Host: Ocellated blenny, *Opinthocentrus ocellatus*, Opisthocentridae.

Locality: Sea of Japan.

Organ: Kidney.

Oocysts: Irregular form, tightly pressed against sporocysts, without residuum.

Sporocysts: Spherical, 4.5–5.0, without residuum.

Reference: [7,30]

***Eimeria dykovae*** Molnár and Rohde, 1988

Host: Red morwong, *Morwong fuscus* (referred as *Cheilodactylus fuscus*), Latridae.

Locality: Tasmania Sea.

Organ: Pyloric caeca, intestine.

Oocysts: Round, 7.8 (7.2–8.4), without residuum (Figure 1(18)).

Sporocysts: Oval, 5.6 (5.1–5.9) × 3.3 (3.2–3.5), with Stieda body, round finely granular residuum.

Sporozoites: Vermiform.

References: [27]

***Eimeria ethmalosae*** Diouf and Toguebaye, 1994

Host: Bonga shad, *Ethmalosa fimbriata*, Clupeidae.

Locality: Off Senegal, Atlantic Ocean.

Organ: Testes.

Oocysts: Spherical, variable size, 22–53 (Figure 1(19)).

Sporocysts: 9.2–12.5 × 6.7–8.3, four to five refringent granular residua, with Stieda body.

This species has three sizes of oocysts which were designated as micro-oocysts, meso-oocysts and macro-oocysts.

References: [16]

***Eimeria etrumei*** Dogiel, 1940

Host: *Etrumeus micropus*, Dussumieriidae.

Locality: Sea of Japan.

Organ: Testes.

Oocysts: Spherical, 33.0–36.0, without residuum (Figure 1(20)).

Sporocysts: Long and fusiform about 20.0 × 5.0, without residuum.

Reference: [7,30]

***Eimeria euzeti*** Daoudi, Radujkovic, Marquès and Bouix, 1987

Host: Common eagle ray, *Myliobatis aquila*, Myliobatidae.

Locality: Kotor Bay, Adriatic Sea.

Organ: Liver.

Oocysts: Spherical 13 (12.5–14.0) without residuum (Figure 1(21)).

Sporocysts: 7.0–8.0 × 5.0–6.0 with numerous refringent granules, without Stieda body.

Sporozoites: 8.0 × 1.5 with a refringent body.

References: [19]

***Eimeria evaginata*** Dogiel 1948

Host: *Sebastes taczanowskii*, Sebastidae; Steller’s sculpin *Myoxocephalus stelleri*, Cottidae.

Locality: Sea of Japan.

Organ: Pyloric caeca.

Oocysts: Spherical 10.0–12.0, without residuum (Figure 1(22)).

Sporocysts: Oval 6.0–6.5 × 4.5, very small residuum.

Sporozoites: Arcuate

Reference: [7,30]

***Eimeria gabonensis*** Diouf and Toguebaye, 1994

Host: Gabon gurnard, *Chelidonichthys gabonensis*, Triglidae.

Locality: Off Senegal, Atlantic Ocean.

Organ: Intestine.

Oocysts: Spherical, 9.6 ± 0.6, without residuum (Figure 1(23)).

Sporocysts: 6.8 ± 0.6 long by 4.6 ± 0.5 wide, Stieda body splitlike, four refringent granular residua.

Sporozoites: Falciform.

References: [16]

***Eimeria gigantea*** (Labbé, 1896) Reichenow, 1921

Syn: *Pfeifferia gigantea* Labbé, 1896, *Coccidium giganteum*, Labbé, 1896, *Pfeifferella gigantea* (Labbé, 1896) Labbé, 1899.

Host: Porbeagle, *Lamna nasus* (referred as *Lamna cornubica*), Lamnidae.

Locality: Off France (Atlantic Ocean or Mediterranean Sea?).

Organ: Spiral valve.

Oocysts: About 70.0 × 40.0.

References: [7,33,34]

***Eimeria gobii*** Fantham, 1932

Host: Barehead goby, *Caffrogobius nudiceps* (referred as *Gobius nudiceps*), Gobiidae.

Locality: Off South Africa (Atlantic Ocean or Indian Ocean?).

Organ: Intestine.

We were unable to obtain the article describing the species or any subsequent one.

Reference: [7]

***Eimeria halleri*** Upton, Bristol, Gardner and Duszynski, 1986

Host: Haller’s round ray, *Urobatis halleri* (referred as *Urolophus halleri*), Urotrygonidae.

Locality: Off Mexico, Pacific Ocean.

Organ: Spiral valve.

Oocysts: Spherical or subspherical, 16.8 (15.0–18.0), without residuum (Figure 1(24)).

Sporocysts: Ovoid, 11.1(10.0–13.0) × 6.8 (6.0–7.5), length/thickness ratio 1.7 (1.4–2.0), with Stieda and substieda bodies. Granular particles or a spherical mass residuum.

Sporozoites: Comma-shaped, 9.9 (9.0–11.0) × 3.2 (2.8–4.0) with ovoid posterior and spherical anterior refractile bodies.

References: [35]

***Eimeria harpodoni*** Setna and Bana, 1935

Host: Bombay-duck, *Harpadon nehereus*, Synodontidae.

Locality: Off Bombay, Indian Ocean.

Organ: Intestine.

Oocysts: Spherical, 14 (12–16) large honeycomb-like residuum (Figure 2(25)).

Sporocysts: Elliptical, 9.6 × 4.6, with a neck and an inverted V-shaped appendage, small granular residuum. 

Sporozoites: Curved.

Reference: [7,13,15]

***Eimeria hexagona*** Lom and Dyková, 1981

Host: Shore rockling, *Gaidropsarus mediterraneus* (referred as *Onos tricirratus*), Gaidropsaridae.

Locality: Banyuls-sur-Mer, Mediterranean Sea.

Organ: Pyloric caeca, intestine.

Oocysts: Average 12, without residuum (Figure 2(26)).

Sporocysts: Ovoid to ellipsoidal 7 (6.8–7.2) × 6 (5.8–6.4), with a collar-like encircling the Stieda body, coarsely granular residuum.

Sporozoites: Sausage-like.

Reference: [7,13,26]

***Eimeria insignis*** Lom and Dyková, 1982

Host: Small red scorpionfish, *Scorpaena notate*, Scorpaenidae.

Locality: Banyuls-sur-Mer, Mediterranean Sea.

Organ: Pyloric caeca.

Oocysts: Spherical, 12 (10–13), without residuum.

Sporocysts: Ellipsoidal 7.0 (6.8–7.4) × 5.2 (4.8–5.4), without Stieda body; no discernible suture indicative of two shell valves, without residuum (Figure 2(27)).

Sporozoites: 4.2 × 1.4.

Reference: [7,22]

***Eimeria ivanae*** Lom and Dyková, 1981

Host: Comber, *Serranus cabrilla,* Serranidae; painted comber, *Serranus scriba*, Serranidae.

Locality: Banyuls-sur-Mer, Mediterranean Sea; Adriatic Sea; off Senegal, Atlantic Ocean.

Organ: Pyloric caeca, intestine.

Oocysts: 10.5 (10.0–11.5), no oocyst residuum (Figure 2(28)).

Sporocysts: 5.8 (5.0–6.5) ×4.4 (4.0–4.6) with Stieda body encircled by a thickening, with granular residuum.

Sporozoites: 8.0 × 6.8.

Reference: [7,16,20,23,26]

***Eimeria kayarensis*** Diouf and Toguebaye, 1994

Host: Brown ray, *Raja miraletus*, Rajidae.

Locality: Off Senegal, Atlantic Ocean.

Organ: Spiral valve.

Oocysts: Ellipsoidal 16.7 ± 1.1 × 13.9 ± 0.9, without residuum (Figure 2(29)).

Sporocysts: Ellipsoidal, with Stieda body, scattered refringent granular residuum.

Sporozoites: Falciform.

Reference: [16]

***Eimeria kotorensis*** Daoudi, Radujkovic, Marquès and Bouix, 1987

Host: Blotched picarel, *Spicara maena*, Sparidae. 

Locality: Kotor Bay, Adriatic Sea.

Organ: Intestine.

Oocysts: Round, 13.0–14.5 (Figure 2(30)).

Sporocysts: Ellipsoids 10.0 (9.5–11.0) × 6.0 (5.0–6.5), with Stieda body and with several central refringent granular residuum.

Sporozoites: Disposed lengthwise, vermiform, 8.5 × 2.0 with refringent granule in a middle position.

Reference: [19]

***Eimeria lairdi*** Lom and Dyková, 1981

Host: Shorthorn sculpin, *Myoxocephalus scorpius*, Cottidae.

Locality: Grand Banks off the coast of New-foundland, Atlantic Ocean.

Organ: Pyloric caeca.

Oocysts: Spherical, 12.0 (10.0–15.0) often coated with a layer of amorphous substance, no residuum (Figure 2(31)).

Sporocysts: Almost spherical, 6.0 (5.0–8.0), with a wide collar-like thickening encircling Stieda body. Central, roughly granular residuum.

Sporozoites: Vermiform 8.0 × 1.5.

Reference: [7,26]

***Eimeria maggieae*** Lom and Dyková, 1981

Host: Common Pandora, *Pagellus erythrinus*, Sparidae.

Locality: Banyuls-sur-Mer, Mediterranean Sea; Adriatic Sea.

Organ: Intestine.

Oocysts: Average 12, wall hardly seen.

Sporocysts: Ellipsoidal to ovoid 5.3 (4.8–5.7) × 8.0 (7.5–8.3), knob-like thickening encircling the Stieda body. Residuum not always visible.

Sporozoites: Twisted.

References: [7,20,23,26]

***Eimeria merlangi*** Zaika, 1966

Host: Whiting, Merlangius merlangus (referred as Odontogadus merlangus euxinus), Gadidae.

Locality: Black Sea.

Organ: Intestine, gall bladder.

We were unable to obtain the article describing this species or any subsequent one.

Reference: [7]

***Eimeria myoxocephali*** Fitzgerald, 1975

Host: Great sculpin, Myoxocephalus poliacanthocephalus, Cottidae.

Locality: US Coast of Washington, Pacific Ocean.

Organ: Intestine.

Oocysts: Spherical 37.2 (34–40), without residuum (Figure 2(32)).

Sporocysts: Small, round residuum.

Sporozoites: Elongate 16.7 × 3.7 sausage-shaped with a single refractile globule.

Reference: [7,36]

***Eimeria nesowai*** Lom and Dyková, 1995

Host: Common silver belly, *Gerres subfasciatus* (referred to as *Gerres ovatus*), Gerreidae.

Locality: Off Australia, Pacific Ocean.

Organ: Pyloric caeca, intestine.

Oocysts: Subspherical, 12.9 (12.0–14.0), without residuum (Figure 2(33)).

Sporocysts: Ellipsoidal, 7.0 (6.5–7.5) × 4.2 (3.5–4.7), with Stieda body, without residuum.

Sporozoites: 6.5 × 1.3.

References: [37]

***Eimeria nishin*** Fujita, 1934

Host: Pacific herring, *Clupea pallasii pallassi*, Clupeidae; Atlantic herring, *Clupea harengus* (referred as *Clupea harengus harengus*), Clupeidae.

Locality: Off Japan, off Russia Far East and off North American coast, Pacific Ocean.

Organ: Testes.

We were unable to obtain the article describing this species or any subsequent one.

Reference: [7]

***Eimeria nucleocola*** Lom and Dyková, 1981

Host: shorthorn sculpin, *Myoxocephalus scorpius*, Cottidae.

Locality: Grand Banks, off the coast of Newfoundland, Atlantic Ocean.

Organ: Pyloric caeca.

Oocysts: 13.0 (12.0–14.0), no residuum (Figure 2(34)).

Sporocysts: Ellipsoidal, 6.8 (6.0–7.3) × 5.3 (4.9–6.0), with knob-like Stieda body hardly visible, without residuum.

Sporozoites: Sausage-like.

References: [7,13,26]

***Eimeria ottojiroveci*** Dyková and Lom, 1983

Syn: *Eimeria jiroveci* Lom and Dyková, 1981 (=nomen pre-occupied)

Host: Thornback ray, *Raja clavata*, Rajidae; Brown ray, *Raja miraletus*, Rajidae.

Locality: Banyuls-sur-Mer, Mediterranean Sea; Adriatic Sea.

Organ: Spiral valve, intestine.

Oocysts: 12.0–13.0, without residuum (Figure 2(35)).

Sporocysts: 8.3 (8.0–8.5) × 6.3 (6.0–6.5), prominent protruding knob-like Stieda body, large granular residuum.

Sporozoites: 11.0 × 1.8, with one pointed and one rounded end.

References: [7,20,23,26]

***Eimeria palavensis*** Marquès and Capapé, 2001

Host: Blackmouth catshark, *Galeus melastomus*, Carcharhiniformes, Pentanchidae.

Locality: Off Languedoc, Mediterranean Sea.

Organ: Spiral valve, intestine.

Oocysts: Round, 12.7 (12.0–14.4), with residuum (Figure 2(36)).

Sporocysts: Tapering toward one end and with Stieda body 10.9 (9.7–12.1) × 5.6 (4.8–6.4), with two refringent granular residua.

Sporozoites: Vermiforms, 9.1 (8.8–9.4) × 2.1 (2.0–2.3).

References: [38]

***Eimeria patagonensis*** Timi and Sardella, 1998

Host: Argentine anchovy, *Engraulis anchoita*, Engraulidae.

Locality: Off Argentina, Atlantic Ocean.

Organ: Testes.

Oocysts: Spherical 43.3 (43.0–44.1), residuum with three to four coarse refringent granules immersed in a thin mass 15.5 (15.1–16.5) × 12.7 (11.9–13.0) (Figure 2(37)).

Sporocysts: Elongated and fusiform, 31.9 (30.9–33.0) × 8.0 (7.2–8.2), without Stieda body, with spherical residuum, 5.1 (4.1–6.2), arranged in a pyramid in the oocyst.

Sporozoites: Ovoid, 10.3 (8.2–12.4) × 5.3 (4.1–6.2).

References: [39]

***Eimeria perciformis*** Diouf and Toguebaye, 1994

Host: Bastard grunt, *Pomadasys incisus*, Haemulidae; Dungat grouper, *Epinephelus goreensis*, Serranidae.

Locality: Off Senegal, Atlantic Ocean.

Organ: Intestine

Oocysts: Spherical, 9.7 ± 0.6, without residuum (Figure 2(38)).

Sporocysts: Ellipsoidal, 5.9 ± 0.8 long by 3.9 ± 0.3 wide, with Stieda body, four refringent granular residua.

Sporozoites: Falciform.

References: [16]

***Eimeria petrovici*** Daoudi, Radujkovic, Marquès and Bouix, 1987

Host: Ocellated wrasse, *Symphodus ocellatus*, Labridae.

Locality: Kotor Bay, Adriatic Sea.

Organ: Intestine.

Oocysts: 12.0 (11.0–12.5), without residuum (Figure 2(39)).

Sporocysts: Ellipsoids, 8.7 (7.5–9.0) × 4.5 (4.0–5.0), with Stieda body and with several central refringent granules.

Sporozoites: 7.5 × 1.5 disposed lengthwise and with a refringent globule and striations in the roundest extremity.

References: [19,23]

***Eimeria phyllopterycis*** Upton, Stamper, Osborn, Mumford, Zwick, Kinsel and Overstreet, 2000

Syn: *Eimeria phyllopteryx* Osborn, Stamper, Reimschuessel, Greenwell, Swick, and Kinsel 1999, *nomen nudum*.

Host: Common seadragon, *Phyllopteryx taeniolatus*, Syngnathidae.

Locality: United States aquarium (endemic to waters off southern Australia).

Organ: Intestine.

Oocysts: Spherical, 30.9 (28.0–34.4), without residuum (Figure 2(40)).

Sporocysts: Ellipsoidal, 24.3 (23.4–25.6) × 10.4 (9.2–11.2), shape index (length/wide) 2.33 (2.14–2.70), with Stieda and substieda bodies, numerous granules of various size residuum.

Sporozoites: 26.0–31.0 × 3.0–3.5, each with 3 refractile bodies.

References: [40]

***Eimeria pleurostici*** Molnár and Rodhe, 1988

Host: *Marilyna pleurosticta* (referred as toad fish, *Sphaeroides pleurosticus*), Tetraodontidae.

Locality: Tasmanian Sea.

Organ: Intestine.

Oocysts: Round, 9.3 (9.1–9.6) (Figure 2(41)).

Sporocysts: Ellipsoidal/oval, 6.7 (6.3–7.0) × 4.4 (4.2–4.6), with plug-like Stieda body, and round or ellipsoidal, finely granular residuum.

Sporozoites: Vermiform.

References: [27]

***Eimeria pneumatophori*** Dogiel, 1948

Host: Chub mackerel, *Scomber japonicus* (referred as *Pneumatophorus japonicus*), Scombridae.

Locality: Sea of Japan.

Organ: Liver.

Oocysts: Spherical, 12.5–13.0, without residuum.

Sporocysts: Oval, 5.0–6.0 ×3.5–4.0; no reference to Stieda body.

References: [7,30]

***Eimeria raiarum*** Van den Berghe, 1937

Syn: *Eimeria rajarum* Diouf and Toguebaye, 1994 *lapsus*.

Host: Blue skate, *Dipturus batis,* (referred as *Raja batis*), Rajidae.

Locality: English Channel and French coast, Atlantic Ocean.

Organ: Intestine.

Oocysts: Spherical 17.5–28.0 with residuum 10.5–2.4.

Sporocysts: Ovoid 7.0 × 6.2 with very small residuum.

References: [7,34]

***Eimeria raibauti*** Daoudi, Radujkovic, Marquès and Bouix, 1989

Host: Poor cod, *Trisopterus minutus*, Gadidae; Norway pout, *Trisopterus esmarkii*, Gadidae.

Locality: Adriatic Sea; Mediterranean Sea; North Sea, Atlantic Ocean.

Organ: Pyloric caeca.

Oocysts: Spherical, 33.0 (30.0–35.0), to ovoid, 37.2 (35–40) × 30.5 (29.0–33.0), without residuum.

Sporocysts: Ovoid, 16.7 (15.5–18.5) × 10.3 (9.0–11.0) with Stieda body and substieda, hexagonal section and refringent granular residuum.

Sporozoites: Vermiforms, 15.0 × 3.5.

References: [23,41,42]

***Eimeria rohdei*** Lom and Dyková, 1995

Host: Fan-bellied leatherjacket, *Monachantus chinensis*, Monachantidae.

Locality: Off Australia, Pacific Ocean.

Organ: Pyloric caeca.

Oocysts: Spherical, 7.5 (7.0–8.5) (Figure 2(42)).

Sporocysts: Ellipsoidal 5.1 (4.5–6.0) × 3.0 (2.5–3.5), fine granular residuum, very small Stieda body.

Sporozoites: Curved, 5.0 × 1.0.

References: [37]

***Eimeria roussillona*** Lom and Dyková, 1981

Host: *Labrus viridis* (referred as *Labrus turdus*), Labridae.

Locality: Banyuls-sur-Mer, Mediterranean Sea.

Organ: Intestine. 

Oocysts: 11.0 (10.0–12.0) (Figure 2(43)).

Sporocysts: Elongate ellipsoids, 7.8 (7.5–8.5) × 4.1 (3.5–4.5), with Stieda body, refractile granular residuum.

Sporozoites: Sausage-like, 10.6 × 1.2.

References: [7,26]

***Eimeria ryptici*** Diouf and Toguebaye, 1994

Host: Spotted soapfish, *Rypticus subbifrenatus*, Serranidae.

Locality: Off Senegal, Atlantic Ocean.

Organ: Intestine.

Oocysts: Spherical, 8.1 ± 0.5, without residuum (Figure 2(44)).

Sporocysts: Ovoid 4.9 ± 0.3 long by 3.4 ± 0.4 wide, Stieda body split-like, three to four refringent granular residua.

Sporozoites: Falciform.

References: [16]

***Eimeria sardinae*** (Thélohan, 1890) Reichenow, 1921

Syn: *Coccidium sardinae* Thélohan, 1890, *Eimeria oxyphila* Dobell, 1919, lapsus, *Eimeria oxyspora* Dobell, 1919, *Eimeria snijdersi* Dobell, 1920.

Host: Atlantic herring, *Clupea harengus* (referred as *Clupea harengus harengus*), Clupeidae; European pilchard, *Sardina pilchardus* (also referred *as Clupea pilchardus*), Clupeidae; round sardinella, *Sardinella aurita*, Clupeidae; madeiran sardinella, *Sardinella maderensis*, Clupeidae; South american pilchard, *Sardinops sagax*, Clupeidae; European sprat, *Sprattus sprattus*, Clupeidae; European anchovy, *Engraulis encrasicolus*, Engraulidae.

Locality: North Sea, Barents Sea; Baltic Sea; Adriatic Sea; Mediterranean Sea; Black Sea; White Sea; Sea of Japan.

Organ: Testes, seminiferous tubules.

Oocysts: Spherical, 33–65 (Figure 2(45)).

Sporocysts: Long, fusiform 25.0–35.0 × 7.0–8.0; ratio of length to width 3:1 or more; large refractile bodies residuum.

Sporozoites: Rod-like.

According to Diouf and Toguebaye [43] this species has three sizes of oocysts which were called micro-oocysts, meso-oocysts and macro-oocysts. 

According to Morrison and Hawkins [44] sporocysts without Stieda body. 

References: [7,13,20,23,30,43,44,45,46,47,48,49,50]

***Eimeria scorpaenae*** Zaika, 1966

Host: Black scorpionfish *Scorpaena porcus*, Scorpaenidae.

Locality: Black Sea.

Organ: Intestine.

We were unable to obtain the article describing this species or any subsequent one.

References: [7]

***Eimeria sillaginis*** Molnár and Rodhe, 1988

Host: Sand sillago (referred as sand whiting), *Sillago ciliata*, Sillaginidae.

Locality: Tasmania Sea. 

Organ: Pyloric caeca, intestine.

Oocysts: Round, 8.0–9.5, without residuum (Figure 2(46)).

Sporocysts: Ellipsoidal, 5.5–7.6 × 3.0–5.0, with Stieda body appearing as

group of 3 small tubercles, round finally granular residuum.

Sporozoites: Vermiform, 4.1–6.0 × 1.5–2.0.

References: [27,37]

***Eimeria smaris*** (Daoudi, 1987) Daoudi, Radujkovic, Marquès and Bouix, 1989

Syn: *Eimeria maenae* Daoudi, 1987.

Host: Picarel, *Spicara smaris*, Sparidae.

Locality: Adriatic Sea.

Organ: Intestine.

Oocysts: Round, 9.2 (8.0–9.5), without residuum (Figure 2(47)).

Sporocysts: Oval, 5.4 (5.0–6.5) × 4.3 (4.0–4.5), with Stieda body, several refringent granular residua.

Sporozoites: Vermiforme 5.0 × 1.5.

References: [20,41]

***Eimeria southwelli*** Halawani, 1930

Syn: *Eimeria quentini* Boulard, 1977

Host: Whitespotted eagle ray, *Aetobatus narinari* (referred as *Aetobatis narinari*), Aetobatidae; cownose ray, *Rhinoptera bonasus*, Rhinopteridae.

Locality: Indian Ocean; Malaysia, Pacific Oceans; North Atlantic Ocean.

Organ: Serosa of the liver, spleen, spiral valve, uterine lining and celomic cavity.

Oocysts: 15–63 × 10–15, polymorphic, pear to elongate shaped, no residuum (Figure 2(48)).

Sporocysts: 10–12 pear-shaped, aligned in a row, with a ridge-like structure along the length and Stieda body. Central residuum.

Sporozoites: Club-shaped, 5.0–10.0 × 2.0.

This species causes high host mortality.

References: [7,13,33,51,52]

***Eimeria sparis*** Sitja-Bobadilla, Palenzuela and Alvarez-Pellitero, 1996

Syn: *Eimeria spari* Diouf and Toguebaye, 1996.

Host: Gilthead seabream, *Sparus aurata*, Sparidae; Bluespotted seabream, *Pagrus caeruleostictus*, Sparidae.

Locality: Mediterranean Sea; Atlantic Ocean.

Organ: Intestine.

Oocysts: Spherical to subspherical, 9.4–14.6, two refractile bodies residuum (Figure 3(49)).

Sporocysts: Ellipsoidal, 6.0–9.7 × 4.0–6.5, with aster shape Stieda body, single body to a mass of granular residuum.

Sporozoites: Vermiform.

References: [6,53,54,55,56]

***Eimeria syacii*** Diouf and Toguebaye, 1994

Host: Channel flounder, *Syacium micrurum*, Cyclopsettidae.

Locality: Off Senegal, Atlantic Ocean.

Organ: Intestine.

Oocysts: Spherical, 10.7 ± 1.1, without residuum (Figure 3(50)).

Sporocysts: Ellipsoidal, 6.6 ± 0.7 long by 4.1 ± 0.5 wide, with Stieda body, with compact residuum.

References: [16]

***Eimeria symphodi*** Daoudi, Radujkovic, Marquès and Bouix, 1989

Host: Sublet, *Symphodus rostratus*, Labridae.

Locality: Adriatic Sea; Mediterranean Sea.

Organ: Intestine.

Oocysts: Round 17.3 (16.0–19.0), without residuum (Figure 3(51)).

Sporocysts: Oval 12.1 (11.0–13.00) × 5.8 (5.5–6.3) with Stieda body.

Sporozoites: Vermiforms, 9.0 × 2.0; two large refringent granular residua.

References: [20,23,41]

***Eimeria syngnathi*** Yakimoff and Gousseff, 1936

Host: Black-stripped pipefish, *Syngnathus abaster* (referred as *Syngnathus nigrolineatus*), Syngnathidae.

Locality: Black Sea; Caspian Sea.

Organ: Intestine.

Oocysts: Ellipsoidal, 24.5–32.0 ×16.7–24.5, with residuum (Figure 3(52)).

Sporocysts: Ellipsoidal, 10.6–14.4 × 7.7–9.2, no reference to Stieda body.

Sporozoites: Broad clavate.

References: [7,30]

***Eimeria triglae*** Daoudi, Radujkovic, Marquès and Bouix, 1989

Host: Tub gurnard, *Chelidonichthys lucerna* (referred as *Trigla lucerna*), Triglidae; piper gurnard, *Trigla lyra*, Triglidae.

Locality: Off France, Mediterranean Sea.

Organ: Pyloric caeca.

Oocysts: Round, 9.8–19.5 (Figure 3(53)).

Sporocysts: Oval, 7.3 (7.0–8.0) × 5.0 (4.5–5.5), hexagonal section and with Stieda body.

Sporozoites: Elongated and with four to six refringent granular residua.

References: [41]

***Eimeria variabilis*** (Thélohan, 1893) Reichenow, 1921

Syn: *Coccidium variabile*, Thélohan, 1893; *Goussia variabilis* (Thélohan, 1893), Labbé, 1896.

Host: Longspined bulhead, *Taurulus bubalis* (referred as *Cottus bubalis*), Cottidae; corkwing wrasse, *Symphodus melops* (referred as *Crenilabrus melops*), Labridae; rock goby, *Gobius paganellus* (referred as *Gobius bicolor*), Gobiidae.

Locality: Celta and Irish Seas, Atlantic Ocean.

Organ: Pyloric caeca, intestine.

Oocysts: Spherical, 11.9–14.6, to subspherical 13.9–14.3 × 9.2–10.9, without residuum (Figure 3(54)).

Sporocysts: 8.5–9.2 × 5.0–5.5, a lid-like Stieda body, without residuum.

Sporozoites: Elongated with both ends flexed.

GenBank accession number: 18S rRNA: GU479674.

References: [7,13,32,57]

***Eimeria zygaenae*** Mandal and Chakravarty, 1965

Host: Winghead shark, *Euphyra blochii* (referred as *Sphyrna blochii*), Sphyrnidae.

Locality: Indian Ocean.

Organ: Intestine

Oocysts: 12.1–14.3.

Sporocysts: About 8.8 × 5.5.

References: [7]

***Epieimeria isabellae*** Lom and Dyková, 1982

Host: European conger, *Conger conger*, Congridae.

Locality: Adriatic Sea; Mediterranean Sea.

Organ: Intestine.

Oocysts: Irregular round, average 12.5 (12–14), without residuum (Figure 3(55)).

Sporocysts: Ovoid, 8.0 (7.6–8.4) × 5.5 (5–6) with collar-like apical thickening around Stieda body, lens-like substieda body, some with a single minute granular residuum.

Sporozoites: Twisted cross-wise or C-shaped, 1.5–2.0.

References: [7,13,20,22,23]

***Epieimeria lomae*** Daoudi, Radujkovic, Marquès and Bouix, 1987

Host: Black scorpionfish, *Scorpaena porcus*, Scorpaenidae.

Locality: Kotor Bay, Adriatic Sea.

Organ: Pyloric caeca.

Oocysts: Spherical, 11.5 (10.0–12.0), without residuum (Figure 3(56)).

Sporocysts: Elliptic, 6.8 (6.5–7.5) × 4.7 (4.0–5.0), very small Stieda body and three to five retractile granular residua.

Sporozoites: Club shaped.

References: [19,20,23]

***Epieimeria ocellata*** Landsberg, 1993

Host: Red drum, *Sciaenops ocellatus*, Sciaenidae.

Locality: Off Florida, Atlantic Ocean.

Organ: Intestine.

Oocysts: Roughly spherical, 9.6 (8.8–11.0) × 9.3 (8.0–11.0), without residuum (Figure 3(57)).

Sporocysts: Ellipsoidal, 6.9 (6.0–8.0) × 4.1 (4.0–5.0), with Stieda body.

Sporozoites: 5.6 (5.0–7.0) × 1.8 (1.0–2.0), with flexed ends.

References: [58]

***Epieimeria puytoraci*** Daoudi, Radujkovic, Marquès and Bouix, 1989

Host: East Atlantic peacock wrasse, *Symphodus tinca*, Labridae.

Locality: Adriatic Sea.

Organ: Intestine.

Oocysts: Round, 13.7 (13.0–14.5), four to seven refringent and spherical residua (Figure 3(58)).

Sporocysts: Ellipsoidal 8.9 (8.5–10.0) × 5.0 (4.5–5.5), with Stieda body and several refringent granular residuum.

Sporozoites: 7.0 × 1.5.

References: [41]

***Goussia aculeati*** Jastrzebski, 1984

Host: Three-spined stickleback, *Gasterosteus aculeatus*, Gasterosteidae.

Locality: Baltic Sea, Atlantic Ocean.

Organ: Intestine.

Oocysts: Subspherical, 14.5 × 11.0 (Figure 3(59)).

References: [13]

***Goussia arrawarra*** Molnár and Rodhe, 1988

Host: Sand sillago (referred as sand whiting), *Sillago ciliata*, Sillaginidae.

Locality: Tasmania Sea.

Organ: Intestine.

Oocysts: Ellipsoidal, 14.5 (14.3–15.1) × 10.7 (10.1–10.9), without residuum (Figure 3(60)).

Sporocysts: Elongate ellipsoidal, 9.4 (9.3–9.6) × 4.8 (4.6–5.0), lentiform or scattered, finely granular residuum.

Sporozoites: Banana-shaped.

References: [27]

***Goussia auxidis*** (Dogiel, 1948) Dyková and Lom, 1983

Syn: *Eimeria auxidis*, Dogiel, 1948.

Host: Pacific saury, *Colobatis saira*, Scomberesocidae; albacore, *Thunnus alalunga*, Scombridae; slender tuna, *Allothunnus fallai*, Scombridae; skipjack tuna, *Katsuwonus pelamis*, Scombridae; yellowfin tuna, *Thunnus albacares*, Scombridae; bullet tuna, *Auxis rochei* (referred as *Auxis maru*), Scombridae; Blue mackerel, *Scomber australasicus*, Scombridae.

Locality: Pacific Ocean.

Organ: Kidney, liver, spleen.

Oocysts: 17–37, without residuum (Figure 3(61)).

Sporocysts: 9–14 × 5–8, no Stieda body.

Sporozoites: Elongate and curve.

References: [7,30,59]

***Goussia bayae*** Matsche, Adams and Blazer, 2019

Host: White perch, *Morone americana*, Moronidae.

Locality: Chesapeake Bay, Atlantic Ocean.

Organ: Hepatic bile ducts and gallbladder. 

Oocysts: Subspherical, 26.2 (22.0–30.0) × 21.8 (18.0–25.0), lenght/width ratio 1.2 (1.1–1.3), with micropyle, without residuum (Figure 3(62)).

Sporocysts: Ellipsoidal, 12.6 (10.0–14.0) × 7.8 (6.0–9.0), lenght/width ratio 1.6 (1.4–1.9), 2 valves joined by a longitudinal suture, without residuum.

Sporozoites: Sligthy arcuate.

GenBank accession number: 18S rRna: MH758783-4; 28S rRNA: MH758782; Cytochrome oxidase I: MH792860, Cytochrome b: MH792861, Cytochrome III: MH792862.

References: [60]

***Goussia bigemina*** Labbé, 1896

Syn: *Eimeria bigemina* (Labbé, 1896) Yakimoff, 1929.

Host: Small sandeel, *Ammodytes tobianus*, Ammodytidae.

Locality: Atlantic Ocean.

Organ: Intestine.

We were unable to obtain the article describing this species or any subsequent one.

References: [7]

***Goussia caseosa*** Lom and Dyková, 1982

Host: Roughhead grenadier, *Macrourus berglax*, Macrouridae.

Locality: North-western Atlantic Ocean.

Organ: Swim bladder, gall bladder, intestine, blood vessels of mesentery.

Oocysts: Round rectangular, 42.0 (40.0–47.0), without residuum (Figure 3(63)).

Sporocysts: Ellipsoid 19.2 (18.0–20.3) × 13.6 (12.0–15.5), thick wall with two valves fused by a faintly visible suture, without residuum.

Sporozoites: C shaped 26.0 × 7.0.

References: [7,13,22,61]

***Goussia clupearum*** (Thélohan, 1894) Labbé, 1896

Syn: *Coccidium* sp., Thélohan, 1892; *Coccidium clupearum*, Thélohan, 1894; *Eimeria clupearum*, (Thélohan, 1894) Doflein, 1909; *Eimeria wenyoni*, Dobell, 1919.

Host: Pontic shad, *Alosa immaculata*, Clupeidae; Atlantic herring, *Clupea harengus*, Clupeidae; Pacific herring, *Clupea pallassii pallassii*, Clupeidae; European pilchard, *Sardina pilchardus* (also referred as *Clupea pilchardus*), Clupeidae; round sardinella, *Sardinella aurita*, Clupeidae; madeiran sardinella, *Sardinella maderensis*, Clupeidae; *Sprattus sprattus*, Clupeidae; European anchovy, *Engraulis encrasicolus*, Engraulidae; *Etrumeus micropus*, Dussumieriidae; Atlantic mackerel, *Scomber scombrus*, Scombridae; Atlantic chub mackerel, *Scomber colias*, Scombridae; Bullet tuna, *Auxis rochei,* Scombridae; little tunny, *Euthynnus alleteratus*, Scombridae; European spratblue whiting, *Micromesistius poutassou*, Gadidae; pouting, *Trisopterus luscus*, Gadidae; False scad, *Caranx rhonchus*, Carangidae; lookdown, *Selene vomer*, Carangidae; Atlantic horse mackerel, *Trachurus trachurus*, Carangidae; common two banded seabream, *Diplodus vulgaris*, Sparidae; two-banded seabream, *Diplodus prayensis*, Sparidae; red porgy, *Pagrus pagrus* (referred as *Sparus pagrus pagrus*), Sparidae; blackspot picarel, *Spiraca melanurus*, Sparidae; West African goatfish, *Pseudopeneus prayensis*, Mullidae; garfish, *Belone belone*, Belonidae.

Locality: Adriatic Sea; Mediterranean Sea; Atlantic Ocean; Pacific Ocean. 

Organ: Liver, gonads, stomach, intestine.

Oocysts: Spherical, 14.0–30.0, suture of the two valves difficult to discern (Figure 3(64)).

Sporocysts: Ellipsoidal, 8.0–12.0 × 4.0–10.0, coarse refractile granular residuum.

Sporozoites: Curved.

GenBank accession number: 18S rRNA: KT025255-56; MW006822-29; MT463280-85; MF468299-307; MF468309-313.

Considerable potential to affect the commercial value of very affected fish.

According to Xavier et al. [62,63], morphologically similar *Goussia* sequenced from fish hosts from family Clupeidae (Atlantic herring and European pilchard) and those sequenced from Gadidae (*T. luscus* and *M. poutassou*) clustered in two distinct clades, and those sequenced from the Atlantic chub mackerel (*Scombridae*) formed a different lineage.

References: [7,13,18,20,23,30,44,46,48,64,65,66,67,68,69,70]

***Goussia cruciata*** (Thélohan, 1892) Labbé, 1896

Syn: *Coccidium cruciatum* Thélohan, 1892; *Eimeria cruciata* (Thélohan, 1892) Yakimoff, 1929.

Host: Atlantic horse mackerel, *Trachurus trachurus* (also referred as *Caranx trachurus*), Carangidae; Blue jack mackerel, *Trachurus picturatus*, Carangidae; Mediterranean horse mackerel, *Trachurus mediterraneus*, Carangidae; Chilean jack mackerel, *Trachurus murphyi*, Carangidae; Cape horse mackerel, *Trachurus capensis*, Carangidae; Cunene horse mackerel, *Trachurus trecae***,** Carangidae; Rough scad, *Trachurus lathami*, Carangidae; White trevally, *Pseudocaranx dentex*, Carangidae.

Locality: Atlantic Ocean; Mediterranean Sea, Adriatic Sea; Pacific Ocean.

Organ: Liver.

Oocysts: Spherical, 17–26, without residuum (Figure 4(65)).

Sporocysts: Ellipsoidal, 7.0–10.0 × 5.5–8.4, two valves connected by a difficult to discern sutural line. Cluster of coarse refractile granular residuum, forming trirradiate star rarely a cross inside the oocyst.

Sporozoites: Vermiforms, 5.0–9.0 × 1.3–1.5.

References: [7,20,23,37,65,68,71,72,73,74,75]

***Goussia dakarensis*** Diouf and Toguebaye, 1993

Host: Parrot grunt, *Pomadasys perotaei*, Haemulidae; bigeye grunt, *Brachydeuterus auritus*, Haemulidae; lesser African threadfin, *Galeoides decadactylus*, Polynemidae.

Locality: Off Senegal, Atlantic Ocean.

ORGAN: Liver.

Oocysts: Spherical 15.0 (13.0–17.0), without residuum (Figure 4(66)).

Sporocysts: Ellipsoid, 8.0 (7.5–9.5) × 6.9 (6.0–7.5), with sutural line and few granular residua.

Sporozoites: Falciform.

References: [66]

***Goussia decapteri*** Diouf and Toguebaye, 1993

Host: False scad, *Caranx rhonchus* (referred as *Decapterus rhonchus*), Carangidae.

Locality: Off Senegal, Atlantic Ocean.

Organ: Liver.

Oocysts: Spherical, 16.1 (13.0–18.5), without residuum (Figure 4(67)).

Sporocysts: Ellipsoidal 7.9 (7.0–8.5) × 5.9 (5.0–7.0), arranged two in two perpendicular planes, single compact mass residuum.

Sporozoites: Falciform.

References: [66]

***Goussia echinata*** Friend, Lovy and Hershberger, 2016

Host: Atlantic herring, *Clupea harengus*, Clupeidae.

Locality: NW Atlantic Ocean.

Organ: Pyloric caeca, intestine.

Oocysts: Ellipsoidal, 18.7 (18.0–19.3) × 11.1 (9.4–11.7); lenght/width 1.7 (1.5–2.0), without residuum, exogened sporulated oocysts with 3 variable long spines in each pole, 15.1 (2.9–20.8) (Figure 4(68)).

Sporocysts: 9.2 (7.8–11.1) × 4.1 (2.9–4.8); lenght/width 2.3 (1.9–3.8), with plentiful residuum.

References: [70]

***Goussia emissolei*** Diouf and Toguebaye, 1993

Host: Barbeled houndshark, *Leptocharias smithii*, Leptochariidae.

Locality: Off Senegal, Atlantic Ocean.

Organ: Intestine. 

Oocysts: Ellipsoidal 19.3 (16.0–22.0) × 15.2 (11.5–18.0), without residuum (Figure 4(69)).

Sporocysts: 9.8 (9.0–12.0) × 7.5 (6.5–8.5), thin sutural line, few refringent granular residua.

Sporozoites: Falciform.

References: [66]

***Goussia ethmalotis*** Obiekezie, 1986

Host: Bonga shad, *Ethmalosa fimbriata*, Clupeidae.

Locality: Off Nigeria, Atlantic Ocean.

Organ: Liver.

Oocysts: Spherical 30.0–37.0, whitout residuum (Figure 4(70)).

Sporocysts: 12.0 (10.0–13.0) × 6.5 (6.0–8.0), with dehiscence suture line, without residuum.

Sporozoites: Reflexed at both ends, arranged crosswise like a “x”.

References: [76]

***Goussia exoceti*** Diouf and Toguebaye, 1993

Host: Fourwing flyingfish, *Hirundichthys affinis*, Exocoetidae.

Locality: Off Senegal, Atlantic Ocean.

Organ: Liver.

Oocysts: Irregular in shape, often ellipsoidal, 26.1 (23.0–30.0) × 22.9 (22.0–25.0), without residuum (Figure 4(71)).

Sporocysts: Ellipsoidal, 12.3 (11.0–14.5) × 8.7 (7.5–9.5), few refringent granular residua.

Sporozoites: Falciform.

References: [66]

***Goussia floridana*** Landsberg, 1993

Host: Red drum, *Sciaenops ocellatus*, Sciaenidae.

Locality: Off Florida, Atlantic Ocean.

Organ: Pyloric caeca, intestine.

Oocysts: Subspherical, 19.9 (19.0–21.0) × 15.9 (14.0–18.0), without residuum (Figure 4(72)).

Sporocysts: Ellipsoidal, 12.6 (11.0–14.0) × 7.5 (7.0–9.0) indistinct suture line, 1 to 14 granular residua.

Sporozoites: 11.0 (10.0–12.0) × 3.9 (3.0–4.0).

References: [58]

***Goussia gadi*** (Fiebiger, 1913) Dyková and Lom, 1981

Syn: *Eimeria gadi* Fiebiger, 1913; *Eimeria* (*Goussia*) *gadi* Fiebiger, 1913; *Eimeria* (*Goussia*) *gadi* (Fiebiger, 1913) Grassé, 1953; *Eimeria jadviagae* Grabda, 1983.

Host: Fourbeard rockling, *Enchelyopus cimbrius*, Gaidropsaridae; haddock, *Melanogrammus aeglefinus* (referred as *Gadus aeglefinus*), Gadidae; Atlantic cod, *Gadus morhua*, Gadidae; saithe, *Pollachius virens* (referred as *Gadus virens*), Gadidae; haddock, *Melanogrammus aeglefinus*, Gadidae; bigeye grenadier, *Macrourus holotrachys* (refered as *Coryphaenoides holotrachis*), Macrouridae; *Coryphaenoides ferrieri*, Macrouridae.

Locality: Atlantic Ocean.

Organ: Swimbladder.

Oocysts: Spherical, 20.0–33.0 (Figure 4(73)).

Sporocysts: 11.0–15.0 × 7.5–10.0.

Sporozoites: C-shaped 16.0 × 4.0.

Can render a non-functional swimbladder being fatal for the fish.

References: [7,13,30,46,77]

***Goussia girellae*** Kent, Fournie, Snodgrass and Elston, 1988

Host: Opaleye, *Girella nigricans*, Girellidae.

Locality: Californian tide pools, Pacific Ocean.

Organ: Gills, intestine, liver and spleen.

Oocysts: Elongate 24.8 (20.1–28.8) × 14.7 (12.5–16.3), without residuum.

Sporocysts: Ellipsoidal, 8.5 (6.8–12.1) × 4.5 (4.0–5.6), no Stieda body, residuum in form of coarse granules.

Sporozoites: With refractile body near posterior end.

Heavily infected fishes moribund.

References: [13,78]

***Goussia kuehae*** Székely, Borkhanuddin, Shaharom, Embong and Molnár, 2013

Syn: *Eimeria* sp. described by Gibson-Kueh et al. [79,80].

Host: Barramundi (referred as Asian seabass), *Lates calcarifer*, Latidae.

Locality: Malaysian, Vietnamese and Australian marine cultures.

Organ: Intestine. 

Oocysts: Ellipsoidal 37.9 (37.0–40.0) × 29.3 (28.0–30.0), two or three pale round residua, 7.8 (7.0–8.5), and some round or amorphous compact residua, 1.0–3.5 (Figure 4(74)).

Sporocysts: Ellipsoidal, 16.2 (15.2–17.0) × 6.7 (5.7–8.0) located very loosely in the oocyst, with two valves and indistinct longitudinal suture and scattered dots residuum.

Sporozoites: Banana-shaped, 14.7 (14.0–15.7) × 2.6 (2.3–2.7).

GenBank accession number:18S rRna: JF261137-40.

References: [81]

***Goussia luciae*** Lom and Dyková, 1982

Host: Red mullet, *Mullus barbatus*, (referred to as sea mullet), Mullidae.

Locality: Off France, Mediterranean Sea; Adriatic Sea.

Organ: Intestine.

Oocysts: Irregular shape, diameter 10.0 (9.5–10.8), without residuum (Figure 4(75)).

Sporocysts: Ellipsoidal 7.5 (6.8–8.0) × 5.5 (5.0–6.0), wall with two valves fused by a distinct suture line

Sporozoites: 12.0 × 1.3 C or S-shaped, one small residuum.

References: [7,20,22,23]

***Goussia lucida*** (Labbé, 1893) Labbé, 1896

Syn: *Coccidium lucidum*, Labbé, 1893; *Eimeria lucida*, (Labbé, 1893) Reichenow, 1921; *Eimeria scyllii*, (Drago, 1902) Levine and Becker, 1933.

Host: Dusky smooth-hound, *Mustelus canis,* Triakidae; Smooth-hound, *Mustelus mustelus* (referred as *Mustelus vulgaris*), Triakidae; Lesser spotted dogfish, *Scyliorhinus caniculus*, Scyliorhinidae; Nursehound, *Scyliorhinus stellaris* (also referred as *Scyllium catulus* and *Scyllium stellaris*), Scyliorhinidae; Picked dogfish, *Squalus acanthias* (also referred as *Acanthias vulgaris* and *Acanthias achantias*), Squalidae; Longnose spurdog, *Squalus blainvillei*, Squalidae.

Locality: Atlantic Ocean; Adriatic Sea; Mediterranean Sea.

Organ: Intestine.

Oocysts: Spherical, 11.0 (10.5–11.5), without residuum (Figure 4(76)).

Sporocysts: Subspherical, 5.6 (5.0–6.0) × 5.3 (5.0–5.5), without Stieda body, residuum composed of just a few granules.

Sporozoites: 7.2 × 1.4, flexed curled around each other.

References: [7,13,20,22,23,29]

***Goussia lusca*** Gestal and Azevedo, 2006

Host: Pouting, *Trisopterus luscus*, Gadidae.

Locality: North East Atlantic Ocean.

Organ: Liver.

Oocysts: 31.7 (28.8–35.4).

Sporocysts: 13.7 (13.1–14.4) × 9.2 (8.5–9.8), wall with outer multilamellated filamentous extensions.

References: [82]

***Goussia microcanthi*** Molnár and Rodhe, 1988

Host: Stripey, *Microcanthus strigatus*, Microcanthidae.

Locality: Tasmania Sea.

Organ: Intestine.

Oocysts: Oval, 12.2 (11.7–13.5) × 10.9 (10.1–11.7) (Figure 4(77)).

Sporocysts: Elongated ellipsoidal, 11.0 (10.9–11.2) × 4.6 (4.5–4.7), scattered residuum.

Sporozoites: Banana-shaped.

References: [27]

***Goussia motellae*** (Labbé, 1893) Labbé, 1896

Syn. *Coccidium motellae*, Labbé, 1893; *Eimeria motellae*, (Labbé, 1893) Yakimoff, 1929.

*Host*: Three-bearded rockling, *Gaidropsarus vulgaris* (referred as *Motella tricirrata*), Gaidropsaridae.

Locality: Off France, Atlantic Ocean.

Organ: Pyloric caeca and intestine.

We were unable to obtain the article describing this species or any subsequent one.

References: [7]

***Goussia senegalensis*** Faye, 1988

Host: African forktail snapper, *Apsilus fuscus*, Lutjanidae; red Pandora, *Pagellus bellottii*, Sparidae.

Locality: Off Senegal, Atlantic Ocean.

Organ: Liver.

Oocysts: Spherical, 17.4 (15.5–9.0), without residuum (Figure 4(78)).

Sporocysts: Ovoid 9.8 (9.5–10.5) × 8.7 (8.0–9.0), with suture and some longitudinal lines and dense granular residuum.

Sporozoites: Falciform and with a transverse band one-third from posterior end.

References: [66]

***Goussia soumbediounensis*** Diouf and Toguebaye, 1993

Host: Barbeled houndshark, *Leptocharias smithii*, Leptochariidae.

Locality: Off Senegal, Atlantic Ocean.

Organ: Intestine.

Oocysts: Spherical, 11.8 (10.0–14.5), without residuum (Figure 4(79)).

Sporocysts: Spherical 6.8 (5.0–9.5), with suture line, few refringent granular residuum. 

Sporozoites: Falciform, with tips folded over.

References: [66]

***Goussia sparis*** Sitja-Bobadilla, Palenzuela and Alvarez-Pellitero, 1996

Host: Gilthead seabream, *Sparus aurata*, Sparidae.

Locality: Mediterranean Sea; Atlantic Ocean.

Organ: Intestine.

Oocysts: 17.4 (16.0–21.0) × 14.4 (13.0–18.0), without residuum (Figure 4(80)).

Sporocysts: 9.5 (8.6–10.3) × 6.5 (5.7–7.4), with scarcely visible suture line, several granular residuum.

Sporozoites: Vermiforms, 9.3 (9.2–9.7) × 3.2 (3.2–3.5).

References: [6]

***Goussia spraguei*** Morrison and Poyton, 1989

Host: Atlantic cod, *Gadus morhua*, Gadidae; haddock, *Melanogrammus aeglefinus*, Gadidae.

Locality: Off Nova Scotia, Atlantic Ocean.

Organ: Kidney. 

Oocysts: Spherical, average 16.2–16.8.

Sporocysts: 11.5–12.2 × 7.4–7.8, with small residuum.

Sporozoites: Curved.

References: [83,84]

***Goussia squali*** (Fitzgerald, 1975) Lom and Dyková, 1992

Syn: *Eimeria squali*, Fitzgerald, 1975.

Host: Picked dogfish, *Squalus acanthias*, Squalidae.

Locality: US coast of Washington, Pacific Ocean.

Organ: Spiral valve.

Oocysts: Ellipsoidal with pitted wall, 24.0–36.0 × 20.0–29.0, with a large (12.4) residuum (Figure 4(81)).

Sporocysts: Unflexed, about 19.6 × 5.9.

Sporozoites: 13.6 × 2.2 slender spindle-shaped.

References: [7,13,36]

***Goussia thelohani*** Labbé, 1896

Syn. *Coccidium* sp. Thélohan, 1894; *Eimeria thélohani* (Labbé, 1896) Yakimoff, 1929.

Host: *Labrus* sp., Labridae; East Atlantic peacock wrasse, *Symphodus tinca*, Labridae; yellowfin bream, *Acanthopagrus australis* Sparidae; goldlined seabream, *Rhabdosargus sarda*, Sparidae.

Locality: Off France, Atlantic Ocean; Adriatic Sea; off Australia, Pacific Ocean.

Organ: Liver.

Oocysts: Round, 16–23.0, without residuum (Figure 4(82)).

Sporocysts: Ovoid, 8.0–11.5 × 7.5–9.5, sutural line quite visible; with one to six refringent granular residuum.

References: [7,20,23,37]

***Goussia trachinoti*** Diouf and Toguebaye, 1993

Host: Pompani, *Trachinotus ovatus*, Carangidae.

Locality: Off Senegal, Atlantic Ocean.

Organ: Liver.

Oocysts: Spherical, 10.9 (9.5–12.0), without residuum.

Sporocysts: Ovoid, 5.9 (5.5–6.5) × 4.5 (4.0–5.0), with refringent granular residuum (Figure 4(83)).

Sporozoites: Vermiform.

References: [66]

### 3.2. Phylogenetic Analysis

Phylogenetic affinities of marine piscine coccidians are depicted in Supplementary Figures S1–S5. Most of the available sequences belong to *Goussia clupearum* (Figure S1), forming the clupearum group described by Xavier et al. [62]. Many of the unidentified sequences of coccidians infecting marine fish clustered with piscine epicellular *Goussia* and *Eimeria* (Figures S2 and S3). As reported By Xavier et al. [85], most of the coccidians so far sequenced from elasmobranchs (both rays and sharks) were basal to many Eimeriorina species infecting other non-piscine vertebrates (Figure S4). Additionally, two of such sequences were instead basal to piscine *Eimeria* (Figure S3). Finally, two sequences of unidentified Coccidia clustered with nodular *Goussia* mostly sequenced by Rosenthal et al. [4] (Figure S5).

## 4. Discussion

A total of 100 species of coccidians were described and reported from 60 families of marine fishes. Most species have been described from marine teleosts, with only 14 species described from marine elasmonbrachs. While roughly only a handful of marine Coccidia belong to genus *Epieimeria*, *Calyptospora*, and *Crystallospora*, species of the genus *Goussia* and *Eimeria* seem to typically infect marine hosts. 

Most of the *Goussia* and *Eimeria* infecting marine fish species were either only reported in the works where they were described, or have been subsequently reported in only one other study (ca. 65% of the species in each genus). Therefore, it is not surprising that the current knowledge on the geographic distribution of marine Coccidia is so limited. For example, the majority of species of *Goussia* (60%) have only been reported from the Atlantic region, and an important fraction (ca. 16%) have also only been reported from the Pacific. Likewise, the vast majority of species of *Eimeria* have either been reported from the Atlantic region (25%), the Mediterranean (26%), or the Pacfic Ocean (25%). Additionally, species that were more frequently reported, besides having a wider known geographic distribution, usually have more known hosts. For example, *Goussia cruciata*, *Goussia clupearum*, and *Eimeria sardinae* have been reported from the Mediterranean Sea and both the Atlantic and Pacific oceans, thus appearing to be fairly cosmopolitan. The three species also appear to be host generalists, with at least *Goussia clupearum* and *Eimeria sardinae* being known to infect hosts belonging to more than one family, and *Goussia cruciata* being known to infect fish from at least two genera within Carangidae. *Goussia thelohani* was also reported from the Mediterranean Sea and the Atlantic and Pacific oceans from two different fish families, despite only being reported in four studies. *Eimeria southwelli* infecting rays from at least two different families has also been reported from the Mediterranean Sea and the Atlantic and Pacific oceans. These observations lead us to conclude that neither geographic range nor host range are sufficiently studied in the vast majority of marine coccidians. However, it is important to note that the existence of cryptic diversity could be common in widespread coccidians, as was found for *Goussia clupearum* [62,63], for which several closely related lineages were found, with most specific to each host species. It is noteworthy that *G. cluperaum* also exhibits high morphological variability, indicating that it may be a species complex (see overview by Xavier and Saraiva [86] regarding the “clupearum” group). A similar scenario has also been proposed for *G. cruciata,* although genetic data are not available to test this hypothesis [75].

Although marine coccidians began to be described more than one century ago, genetic data on marine fish coccidians have only started to be produced since 2010s, with various genetic sequences presently available in GenBank. However, genetic analysis of marine fish Coccidia has been largely disconnected from the morphological analysis of specimens, and genetic data are only available for six formally described marine coccidian species. Though generally, genetic data are still largely missing for piscine Coccidia (both freshwater and marine), phylogenetic reconstructions based on 18S rRNA permitted raising the hypothesis that Coccidia infecting aquatic organisms are basal to those infecting terrestrial taxa [87], and that *Calyptospora* might have diverged earlier than *Goussia* and *Eimeria* [88]. Current knowledge shows that neither *Eimeria* nor *Goussia* are monophyletic. Specifically, piscine *Goussia* spp. were shown to be polyphyletic with different evolutionary lineages, with the species infecting the gut clustering mostly according to their development (i.e., nodular, dispersed, and epicellular) [4]. *Goussia* infecting other organs (*Goussia leucisci* isolated from the kidney of the roach and *Goussia siliculiformis* sequenced from the gut mesentery of the freshwater bream) were considered to have evolved independently [4]. Furthermore, several studies genetically characterized *Goussia clupearum* (the type species from the genus) from different marine fishes and tissues, indicating that it is possibly a complex of species which are sister taxa to the *Calyptospora* [62,63]. Interestingly, *G. clupearum* and *Calyptospora* exhibit high levels of intraspecific genetic variability [62,63,88].

Our phylogenetic reconstruction, combining all available sequences of marine Coccidia, showed that most unidentified genetic lineages likely belong to *Goussia* (n = 11) and have phylogenetic affinities with epicellular and nodular *Goussia*. Those clustering with epicellular *Goussia* included some Coccidia sequenced from other organs than the gut, with such lineages clustering together. Three sequences retrieved from several tissues of *Pagrus caeruleostictus* formed a basal clade to *Calyptospora*; however, they could correspond to a different *Goussia.* For example, *Goussia clupearum* seems to be closely related to this group. Only four unidentified coccidian sequences clustered within *Eimeria*, one likely being *E. variabilis* (see [89]). Finally, the lineages sequenced from elasmobranchs are the most enigmatic, some being basal to *Eimeria* (n = 2) and others being basal to other Eimeriorina Coccidia (including *Eimeria* and *Schellackia*), infecting marine/freshwater turtles, terrestrial reptiles, birds, and mammals [85]. Unfortunately, none of the Coccidia sequenced from elasmobranchs have been formally identified. However, these patterns suggest that Coccidia infecting this host group comprise older lineages than the ones infecting other vertebrates. 

## 5. Conclusions

Our synopsis shows that the current knowledge regarding marine fish Coccidia is still limited. It seems that a taxonomic gap does exist, exaggerated by a shortage of taxonomists [90]. This is not unexpected due to the innacessible nature of the marine environement which hinders the access to fresh fish tissues which would enable species descriptions and accurate identifications. On the contrary, genetic data are starting to be available for many coccidians infecting marine fish; however, it is necessary to merge the gap between the genetic data being produced and appropriate morphological characterization. It is also worth noting that phylogenetic inferences are currently restricted to information from a single gene (18S rRNA) and that developing new genetic markers that amplify across Coccidia is therefore essential.

## Figures and Tables

**Table 1:** Marine fish species and their reported coccidian species.

	Host Species	Coccidian Species
ELASMOBRANCHII		
Carcharhiniformes		
Leptochariidae	*Leptocharias smithii*	*Goussia emissolei*
		*Goussia soumbediounensis*
Pentanchidae	*Galeus melastomus*	*Eimeria palavensis*
Scyliorhinidae	*Scyliorhinus caniculus*	*Goussia lucida*
	*Scyliorhinus stellaris*	*Goussia lucida*
Sphyrnidae	*Euphyra blochii*	*Eimeria zygaenae*
Triakidae	*Mustelus canis*	*Goussia lucida*
	*Mustelus mustelus*	*Goussia lucida*
Lamniiformes		
Lamnidae	*Lamna nasus*	*Eimeria gigantea*
Myliobatiformes		
Aetobatidae	*Aetobatus narinari*	*Eimeria southwelli*
Myliobatidae	*Myliobatis aquila*	*Eimeria euzeti*
Rhinopteridae	*Rhinoptera bonasus*	*Eimeria southwelli*
Urotrygonidae	*Urobatis halleri*	*Eimeria chollaensis*
		*Eimeria halleri*
Rajiformes		
Rajidae	*Dipturus batis*	*Eimeria raiarum*
	*Raja clavata*	*Eimeria ottojiroveci*
	*Raja miraletus*	*Eimeria kayarensis*
		*Eimeria ottojiroveci*
Squaliformes		
Squalidae	*Squalus acanthias*	*Goussia lucida*
		*Goussia squali*
	*Squalus blainvillei*	*Goussia lucida*
ACTINOPTERI		
Anguilliformes		
Anguillidae	*Anguilla anguilla*	*Eimeria anguillae*
	*Anguilla australis*	*Eimeria anguillae*
	*Anguilla dieffenbachiii*	*Eimeria anguillae*
	*Anguilla rostrata*	*Eimeria anguillae*
Congridae	*Conger conger*	*Epieimeria isabellae*
Atheriniformes		
Atherinidae	*Atherina boyeri*	*Eimeria atherinae*
Atherinopsidae	*Menidia beryllina*	*Calyptospora funduli*
Aulopiformes		
Synodontidae	*Harpadon nehereus*	*Eimeria harpodoni*
Batrachoidiformes		
Batrachoididae	*Opsanus beta*	*Calyptospora funduli*
Beloniformes		
Belonidae	*Belone belone*	*Goussia clupearum*
Exocoetidae	*Hirundichthys affinis*	*Goussia exoceti*
Scomberesocidae	*Colobatis saira*	*Goussia auxidis*
Blenniiformes		
Blenniidae	*Blennius pholis*	*Eimeria dingleyi*
Clinidae	*Clinus superciliosus*	*Eimeria clini*
Carangaria		
Latidae	*Lates calcarifer*	*Goussia kuehae*
Polynemidae	*Galeoides decadactylus*	*Goussia dakarensis*
Carangiformes		
Carangidae	*Caranx rhonchus*	*Goussia clupearum*
		*Goussia cruciata*
		*Goussia decapteri*
	*Pseudocaranx dentex*	*Goussia cruciata*
	*Selene vomer*	*Goussia clupearum*
	*Trachinotus ovatus*	*Goussia trachinoti*
	*Trachurus capensis*	*Goussia cruciata*
	*Trachurus latham*	*Goussia cruciata*
	*Trachurus mediterraneus*	*Goussia cruciata*
	*Trachurus murphyi*	*Goussia cruciata*
	*Trachurus picturatus*	*Goussia cruciata*
	*Trachurus trachurus*	*Goussia clupearum*
		*Goussia cruciata*
	*Trachurus trecae*	*Goussia cruciata*
Centrarchiformes		
Girellidae	*Girella nigricans*	*Goussia girellae*
Latridae	*Morwong fuscus*	*Eimeria cheilodactyli*
		*Eimeria dykovae*
Microcanthidae	*Microcanthus strigatus*	*Goussia microcanthi*
Clupeiformes		
Clupeidae	*Alosa immaculata*	*Goussia clupearum*
	*Brevoortia tyrannus*	*Eimeria brevoortina*
	*Clupea harengus*	*Eimeria nishin*
		*Eimeria sardinae*
		*Goussia clupearum*
	*Clupea pallasii*	*Eimeria nishin*
	*Ethmalosa fimbriata*	*Eimeria ethmalosae*
		*Goussia ethmalotis*
	*Sardina pilchardus*	*Eimeria sardinae*
		*Goussia clupearum*
	*Sardinella aurita*	*Eimeria sardinae*
		*Goussia clupearum*
	*Sardinella maderensis*	*Eimeria sardinae*
		*Goussia clupearum*
	*Sardinops sagax*	*Eimeria sardinae*
	*Sprattus sprattus*	*Eimeria sardinae*
		*Goussia clupearum*
Dussumieriidae	*Etrumeus micropus*	*Eimeria etrumei*
		*Goussia clupearum*
Engraulidae	*Engraulis anchoita*	*Eimeria patagomemsis*
	*Engraulis encrasicolus*	*Eimeria sardinae*
		*Goussia clupearum*
Cyprinodontiformes		
Fundulidae	*Fundulus grandis*	*Calyptospora funduli*
	*Fundulus heteroclitus*	*Calyptospora funduli*
	*Fundulus jenkinsi*	*Calyptospora funduli*
	*Fundulus pulvereus*	*Calyptospora funduli*
	*Fundulus similis*	*Calyptospora funduli*
Eupercaria		
Gerreidae	*Gerres subfasciatus*	*Eimeria nesowai*
Haemulidae	*Brachydeuterus auritus*	*Goussia dakarensis*
	*Pomadasys incisus*	*Eimeria perciformes*
	*Pomadasys perotaei*	*Goussia dakarensis*
Labridae	*Bodianus speciosus*	*Eimeria catalana*
	*Labrus viridis*	*Eimeria roussillona*
	*Symphodus cinereus*	*Eimeria banyulensis*
		*Eimeria catalana*
	*Symphodus mediterraneus*	*Eimeria banyulensis*
		*Eimeria catalana*
	*Symphodus melops*	*Eimeria variabilis*
	*Symphodus ocellatus*	*Eimeria petrovici*
	*Symphodus rostratus*	*Eimeria symphodi*
	*Symphodus tinca*	*Epieimeria puytoraci*
		*Goussia thelohani*
Moronidae	*Dicentrarchus labrax*	*Eimeria bouixi*
		*Eimeria dicentrarchi*
	*Morone americana*	*Goussia bayae*
Scaridae	*Scarus hoefleri*	*Eimeria catalana*
Sciaenidae	*Sciaenops ocellatus*	*Epieimeria ocellata*
		*Goussia floridana*
Sillaginidae	*Sillago ciliata*	*Eimeria ciliatae*
		*Eimeria sillaginis*
		*Goussia arrtawarra*
Sparidae	*Acanthopagrus australis*	*Goussia thelohani*
	*Diplodus prayensis*	*Goussia clupearum*
	*Diplodus vulgaris*	*Goussia clupearum*
	*Pagellus bellottii*	*Eimeria ashburneri*
		*Goussia senegalensis*
	*Pagellus erythrinus*	*Eimeria maggieae*
	*Pagrus caeruleostictus*	*Eimeria ashburneri* *Eimeria sparis*
	*Pagrus pagrus*	*Goussia clupearum*
	*Rhabdosargus sarda*	*Goussia thelohani*
	*Sparus aurata*	*Eimeria sparis*
		*Goussia sparis*
	*Spicara maena*	*Eimeria kotorensis*
	*Spicara smaris*	*Eimeria smaris*
	*Spiraca melanurus*	*Goussia clupearum*
Gadiformes		
Gadidae	*Gadus morhua*	*Goussia gadi*
		*Goussia spraguei*
	*Melanogrammus aeglefinus*	*Goussia gadi*
		*Goussia spraguei*
	*Merlangius merlangus*	*Eimeria merlangi*
	*Micromesistius poutassou*	*Goussia clupearum*
	*Pollachius virens*	*Eimeria gadi*
	*Trisopterus esmarkii*	*Eimeria raibauti*
	*Trisopterus luscus*	*Goussia clupearum*
		*Goussia lusca*
	*Trisopterus minutus*	*Eimeria raibauti*
Gaidropsaridae	*Enchelyopus cimbrius*	*Goussia gadi*
	*Gaidropsarus mediterraneus*	*Crystallospora crystalloides*
		*Eimeria hexagona*
	*Gaidropsarus vulgaris*	*Crystallospora crystalloides*
		*Goussia motellae*
Macrouridae	*Coryphaenoides ferrieri*	*Goussia gadi*
	*Macrourus berglax*	*Goussia caseosa*
	*Macrourus holotrachys*	*Goussia gadi*
Gobiidae	*Caffrogobius nudiceps*	*Eimeria gobii*
	*Gobius paganellus*	*Eimeria variabilis*
Holocentriformes		
Holocentridae	*Sargocentron hastatum*	*Eimeria adioryxi*
Mulliformes		
Mullidae	*Mullus barbatus*	*Goussia luciae*
	*Pseudopeneus prayensis*	*Goussia clupearum*
Perciformes		
Agonidae	*Tilesina gibbosa*	*Eimeria citriformis*
Ammodytidae	*Ammodytes tobianus*	*Goussia bigemina*
Cottidae	*Myoxocephalus poliacanthocephalus*	*Eimeria myoxocephali*
	*Myoxocephalus scorpius*	*Eimeria lairdi*
		*Eimeria nucleocola*
	*Myoxocephalus stelleri*	*Eimeria evaginata*
Gasterosteidae	*Gasterosteus aculeatus*	*Goussia aculeati*
Opisthocentridae	*Opinthocentrus ocellatus*	*Eimeria dogieli*
Scorpaenidae	*Scorpaena notate*	*Eimeria insignis*
	*Scorpaena porcus*	*Eimeria scorpaenae*
		*Epieimeria lomae*
Serranidae	*Epinephelus goreensis*	*Eimeria perciformes*
	*Serranus cabrilla*	*Eimeria ivanae*
	*Serranus scriba*	*Eimeria ivanae*
Triglidae	*Chelidonichthys gabonensis*	*Eimeria gabonensis*
	*Chelidonichthys lucerna*	*Eimeria triglae*
	*Trigla lyra*	*Eimeria triglae*
Pleuronectiformes		
Cyclopsettidae	*Syacium micrurum*	*Eimeria syacii*
Scombriformes		
Scombridae	*Allothunnus fallai*	*Goussia auxidis*
	*Auxis rochei*	*Goussia auxidis*
		*Goussia clupearum*
	*Euthynnus alleteratus*	*Goussia clupearum*
	*Katsuwonus pelamis*	*Goussia auxidis*
	*Scomber australasicus*	*Goussia auxidis*
	*Scomber colias*	*Goussia clupearum*
	*Scomber japonicus*	*Eimeria pneumatophori*
	*Scomber scombrus*	*Goussia clupearum*
	*Thunnus alalunga*	*Goussia auxidis*
	*Thunnus albacares*	*Goussia auxidis*
Syngnathiformes		
Syngnathidae	*Phyllopteryx taeniolatus*	*Eimeria phyllopterycis*
	*Syngnathus abaster*	*Eimeria syngnathi*
Tetraodontiformes		
Monachantidae	*Monachantus chinensis*	*Eimeria rohdei*
Tetraodontidae	*Marilyna pleurosticta*	*Eimeria pleurostici*

## Data Availability

All data are reported in the manuscript.

## Supplementary Materials

The following supporting information can be downloaded at: https://www.mdpi.com/article/10.3390/ani13132119/s1, Figure S1. Bayesian phylogenetic reconstruction of available sequences of *Goussia clupearum*, *Calyptospora* and other unidentified lineages based on 558 bp of the 18S rRNA gene and HKY+G sequence model of evolution. Figure S2. Bayesian phylogenetic reconstruction of available sequences of epicellular *Goussia* and closely related unidentified sequences, based on 508 bp of the 18S rRNA gene and GTR+G sequence model of evolution. Figure S3. Bayesian phylogenetic reconstruction of available sequences of *Eimeria* and closely related unidentified sequences, based on 569 bp of the 18S rRNA gene and GTR+G sequence model of evolution. Figure S4. Bayesian phylogenetic reconstruction of available sequences of Coccidia infecting elasmobranchs based on 515 bp of the 18S rRNA gene, using GTR+G sequence model of evolution. Figure S5. Bayesian phylogenetic reconstruction of available sequences of nodular and dispersed *Goussia* and closely related unidentified sequences, based on 565 bp of the 18S rRNA gene and GTR+G sequence model of evolution.
