# Supplementary material for: Synopsis of the Species of Coccidians Reported in Marine Fish
Source: Animals (Basel). 2023 Jun 26;13(13):2119. doi: 10.3390/ani13132119 (PMC10339986; doi:10.3390/ani13132119)
Supplement: Supplementary file 1 [file animals-13-02119-s001.zip › Supplementary Figure Legends.pdf]

Supplementary Materials: (Figures S1–S5)

Supplementary Figure legends:

Figure S1. Bayesian phylogenetic reconstruction of available sequences of *Goussia clupearum*, *Calyptospora* and other unidentified lineages based on 558 bp of the 18S rRNA gene and HKY+G sequence model of evolution.

Figure S2. Bayesian phylogenetic reconstruction of available sequences of epicellular *Goussia* and closely related unidentified sequences, based on 508 bp of the 18S rRNA gene and GTR+G sequence model of evolution.

Figure S3. Bayesian phylogenetic reconstruction of available sequences of *Eimeria* and closely related unidentified sequences, based on 569 bp of the 18S rRNA gene and GTR+G sequence model of evolution.

Figure S4. Bayesian phylogenetic reconstruction of available sequences of coccidia infecting elasmobranchs based on 515 bp of the 18S rRNA gene, using GTR+G sequence model of evolution.

Figure S5. Bayesian phylogenetic reconstruction of available sequences of nodular and dispersed *Goussia* and closely related unidentified sequences, based on 565 bp of the 18S rRNA gene and GTR+G sequence model of evolution.
