# Supplementary figures and images for: Synopsis of the Species of Coccidians Reported in Marine Fish
Source: Animals (Basel). 2023 Jun 26;13(13):2119. doi: 10.3390/ani13132119 (PMC10339986; doi:10.3390/ani13132119)

JF261140  
*Goussia kuehne*

# BAYESIAN INFERENCE POSTERIOR PROBABILITIES

- > 0.99
- 0.85-0.91
- 0.50-0.73

DQ060661.2

0.04

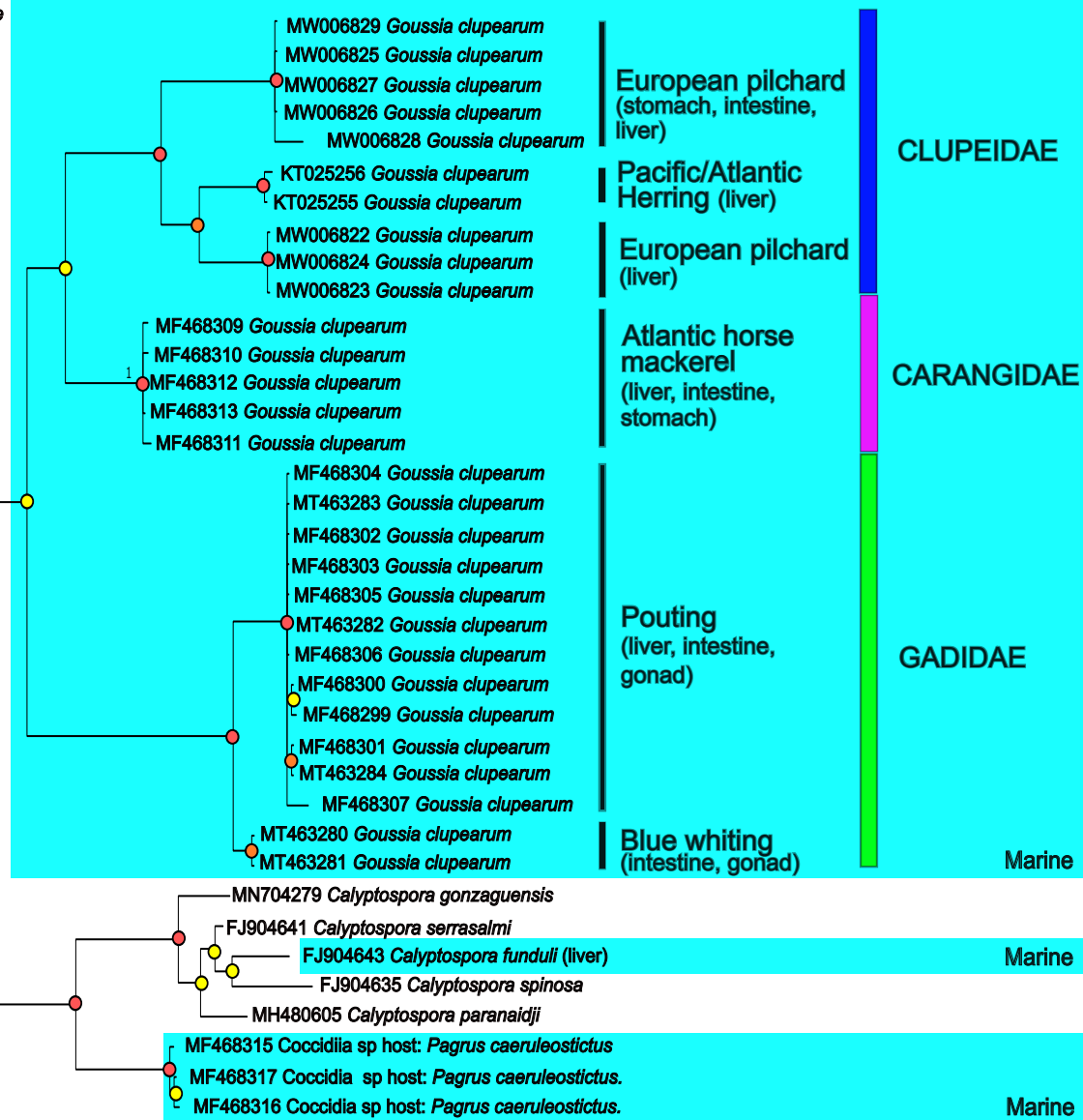

Supplement: Supplementary file 1 [file animals-13-02119-s001.zip › FigS1_final.pdf]

**BAYESIAN INFERENCE  
POSTERIOR PROBABILITIES**

- > 0.98
- 0.90 - 0.98
- 0.60 - 0.67

Leucisci type

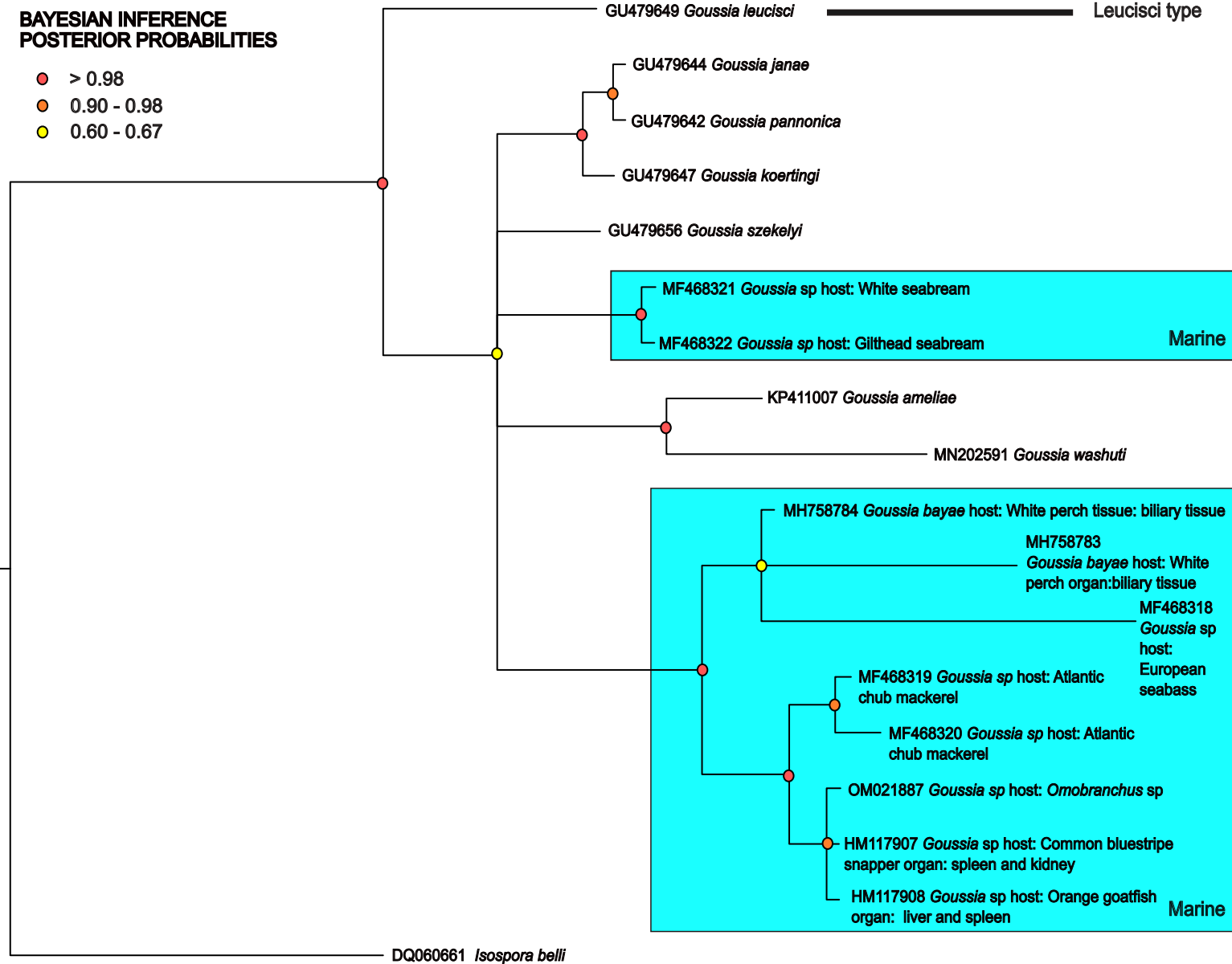

Supplement: Supplementary file 1 [file animals-13-02119-s001.zip › FigS2_final.pdf]

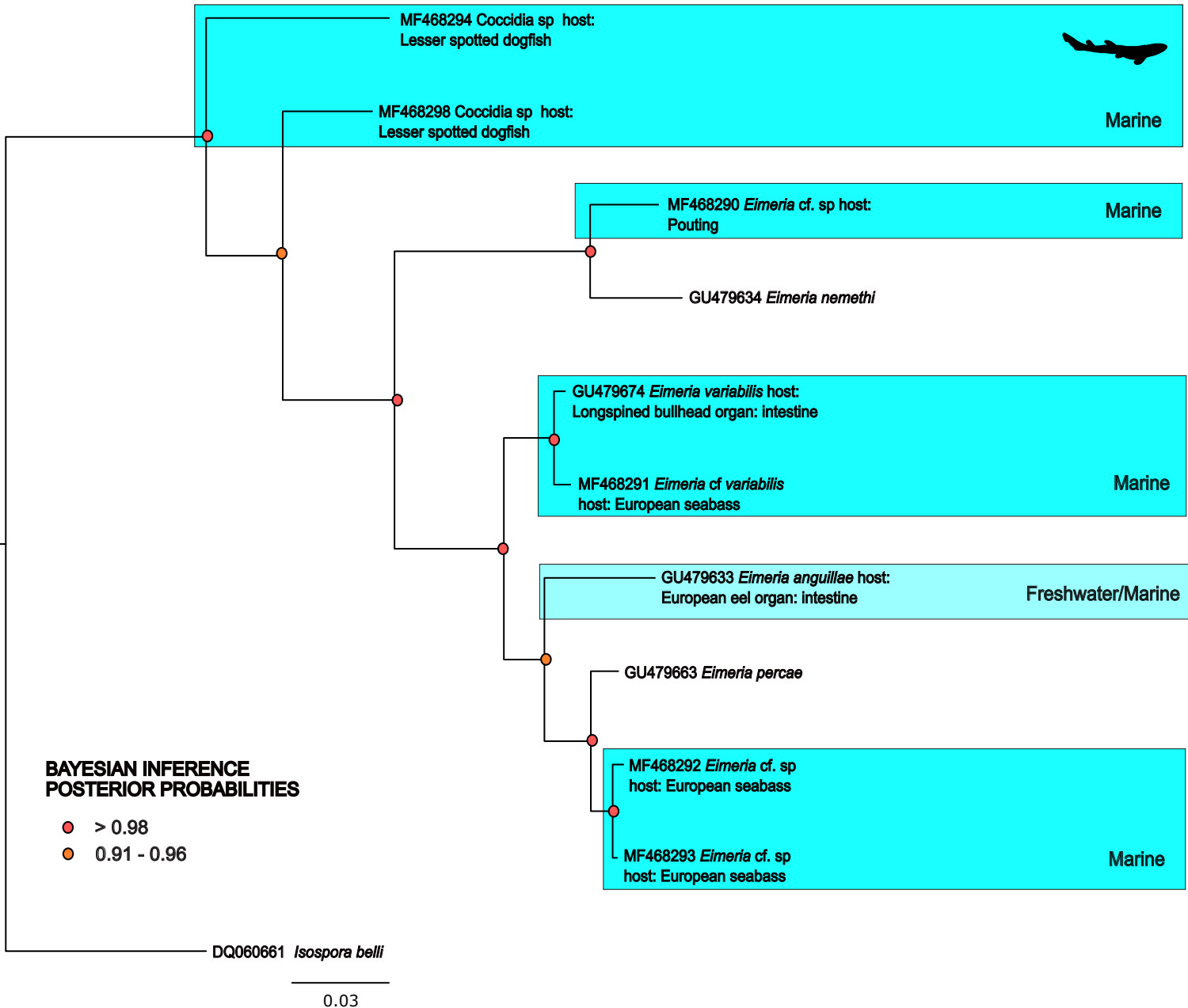

Supplement: Supplementary file 1 [file animals-13-02119-s001.zip › FigS3_final.pdf]

# BAYESIAN INFERENCE POSTERIOR PROBABILITIES

- > 0.98
- 0.89-0.94
- 0.53

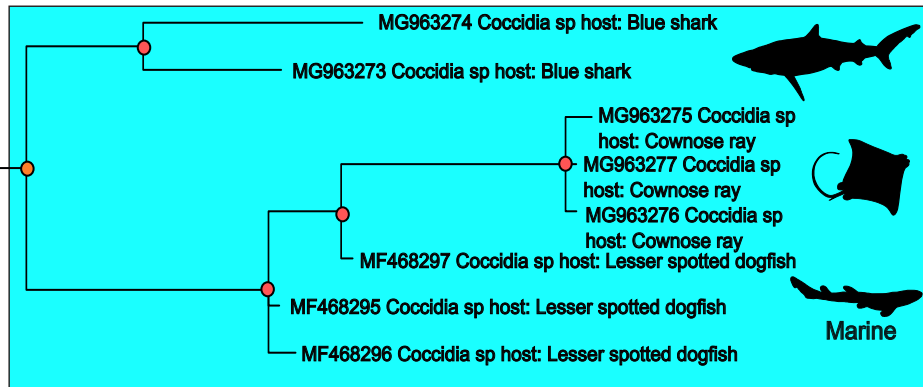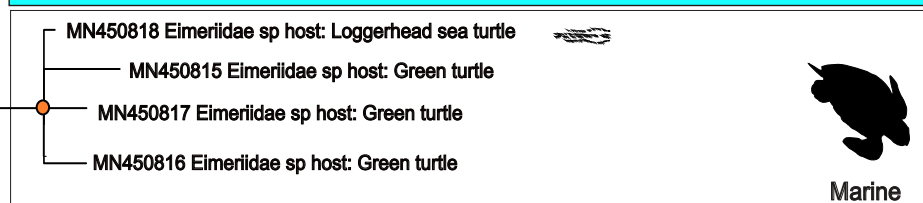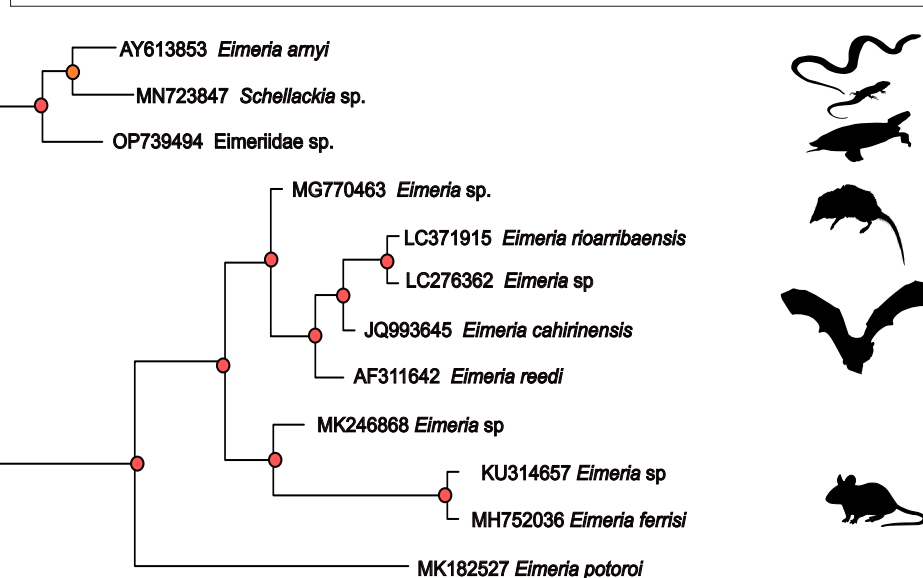

AY728896 Intranuclear coccidium

0.02

Supplement: Supplementary file 1 [file animals-13-02119-s001.zip › FigS4_final.pdf]

NODULAR

BAYESIAN INFERENCE  
POSTERIOR PROBABILITIES

- > 0.98
- 0.70 -0.74
- 0.55-0.59

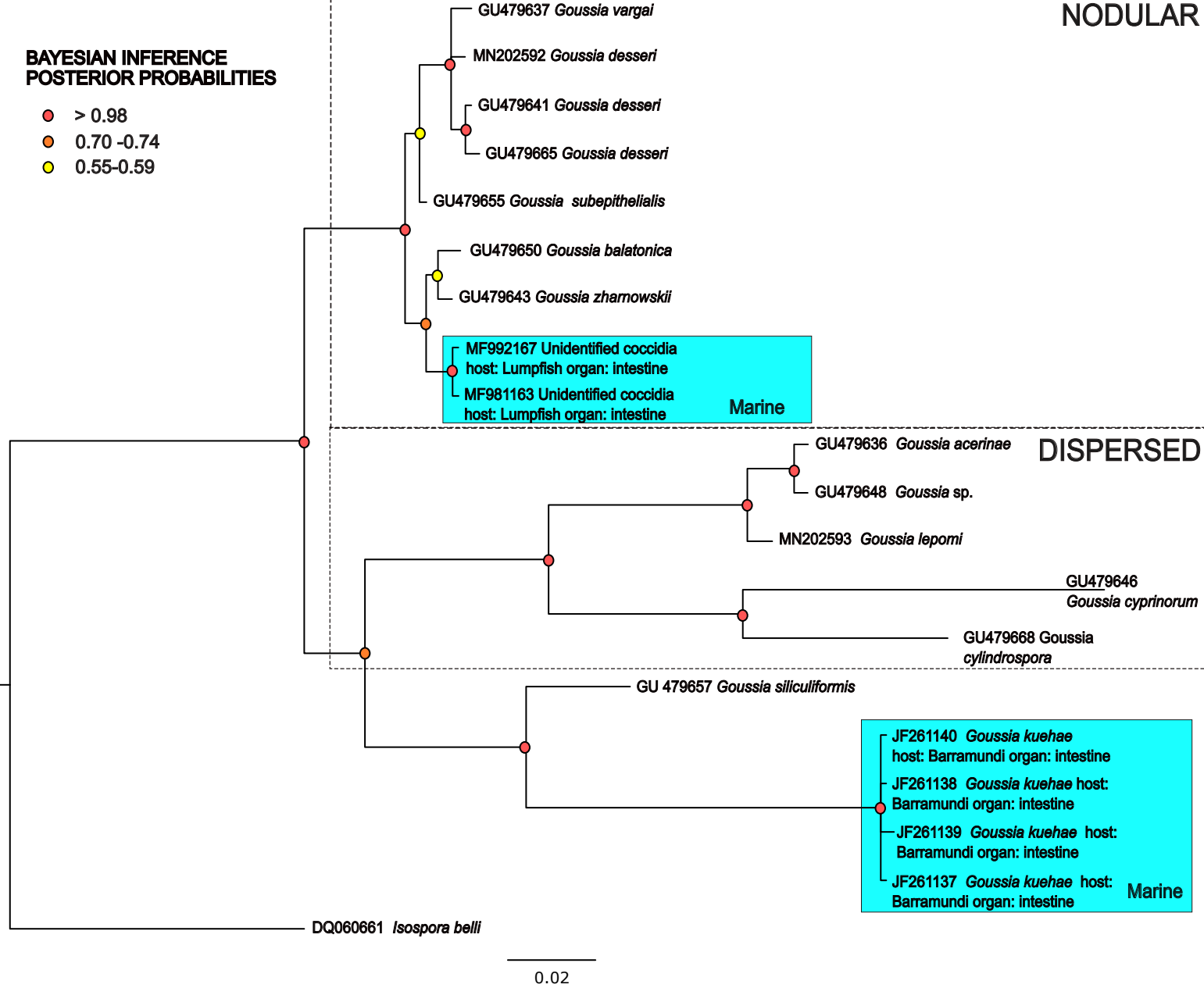

Supplement: Supplementary file 1 [file animals-13-02119-s001.zip › FigS5_final.pdf]
